# Analysis of the Secretomes of *Paracoccidioides* Mycelia and Yeast Cells

**DOI:** 10.1371/journal.pone.0052470

**Published:** 2012-12-18

**Authors:** Simone Schneider Weber, Ana Flávia Alves Parente, Clayton Luiz Borges, Juliana Alves Parente, Alexandre Melo Bailão, Célia Maria de Almeida Soares

**Affiliations:** Laboratório de Biologia Molecular, Instituto de Ciências Biológicas, Universidade Federal de Goiás, Goiânia, Goiás, Brazil; Geisel School of Medicine at Dartmouth, United States of America

## Abstract

*Paracoccidioides*, a complex of several phylogenetic species, is the causative agent of paracoccidioidomycosis. The ability of pathogenic fungi to develop a multifaceted response to the wide variety of stressors found in the host environment is important for virulence and pathogenesis. Extracellular proteins represent key mediators of the host-parasite interaction. To analyze the expression profile of the proteins secreted by *Paracoccidioides*, *Pb*01 mycelia and yeast cells, we used a proteomics approach combining two-dimensional electrophoresis with matrix-assisted laser desorption ionization quadrupole time-of-flight mass spectrometry (MALDI-Q-TOF MS/MS). From three biological replicates, 356 and 388 spots were detected, in mycelium and yeast cell secretomes, respectively. In this study, 160 non-redundant proteins/isoforms were indentified, including 30 and 24 proteins preferentially secreted in mycelia and yeast cells, respectively. *In silico* analyses revealed that 65% of the identified proteins/isoforms were secreted primarily via non-conventional pathways. We also investigated the influence of protein export inhibition in the phagocytosis of *Paracoccidioides* by macrophages. The addition of Brefeldin A to the culture medium significantly decreased the production of secreted proteins by both *Paracoccidioides* and internalized yeast cells by macrophages. In contrast, the addition of concentrated culture supernatant to the co-cultivation significantly increased the number of internalized yeast cells by macrophages. Importantly, the proteins detected in the fungal secretome were also identified within macrophages. These results indicate that *Paracoccidioides* extracellular proteins are important for the fungal interaction with the host.

## Introduction

The *Paracoccidioides* genus represents the causative agent of paracoccidioidomycosis (PCM), one of the most frequent systemic mycoses that affect rural populations in Latin America [1]. The genus comprises four phylogenetic lineages (S1, PS2, PS3 and *Pb*01-like) [2], [3]. The phylogenetic analysis of many *Paracoccidioides* isolates has resulted in the differentiation of the genus into two species, *P. brasiliensis*, which represents a complex of three phylogenetic groups and *P. lutzii*, which includes the Pb01-like isolate [4], [5].

*Paracoccidioides* grows as a yeast form in the host tissue and in culture at 36°C, while it grows as mycelium in the saprobic condition and in culture at room temperature (18–23°C) [6]. As the dimorphism is dependent on temperature, when the mycelia/conidia are inhaled into the host lungs, the transition of the mycelia to the pathogenic yeast phase occurs [7].

The ability of the pathogenic fungi to develop a multifaceted response to the wide variety of stressors found in the host environment is of extreme importance for the virulence and pathogenesis [8]. Many of those molecules are extracellular factors, which are either secreted or associated with the fungal cell wall. The secreted proteins perform important functions, such as the provision of nutrients, cell-to-cell communication, and detoxification of the environment and the killing of potential competitors [9]–[11].

In eukaryotic cells, the classical secretory pathway of proteins is driven by a canonical N-terminal signal peptide. This classical pathway involves the recognition of a signal sequence in the proteins to be exported, which results in their translocation across the endoplasmic reticulum (ER) membrane and delivery to the Golgi apparatus [12]. Functional proteins lacking predicted signal peptides are secreted into the extracellular medium, thereby suggesting the existence of unconventional mechanisms of protein secretion in eukaryotes [9], [13], [14]. A repertoire of hypothetical mechanisms for driving proteins that lack an N-terminal secretion signal through the plasma membrane to the outside of the cell has been described for *Candida albicans* and *Saccharomyces cerevisiae*. These mechanisms include: passive transport, translocation, substrate-specific recognition and the affinity of some proteins to secretory vesicles, which lead to adhesion or internalization in endosomal sub compartments [9]. In this later mechanism, which is described as nonconventional export, the formation of the exosomes is required, which involves vesicles derived from membrane invagination (endosomes) resulting in the release of internal vesicles to the extracellular environment [15]–[17]. Studies have demonstrated that several fungi produce extracellular vesicles containing key molecules associated with virulence, stress response and vesicular transport [18]–[20]. The extracellular vesicles produced by *Cryptococcus neoformans* and *Histoplasma capsulatum* are used for the delivery of molecules associated with pathogenesis to the extracellular space. This group of molecules includes well-known virulence factors, such as enzymes associated with capsule synthesis in *C. neoformans*, laccase, acid phosphatase, heat shock proteins and several antioxidant proteins, including superoxide dismutase, thioredoxin and catalases. These proteins are recognized by the sera of patients with cryptococcosis and histoplasmosis, thereby suggesting that these proteins are produced during human infection [18], [19]. Additionally, the co-incubation of cryptococcal extracellular vesicles with murine macrophages results in a dose-dependent stimulation of nitric oxide production by phagocytes and an increase in extracellular tumor necrosis factor alpha (TNF-α), interleukin-10 (IL-10) and transforming growth factors β (TGF-β) levels [21]. These findings indicate that the extracellular vesicles of *C. neoformans* are biologically active and can stimulate macrophage function, thereby activating these phagocytic cells to enhance their antimicrobial activity. Taken together, these data suggest that fungal secretory vesicles possess the potential to influence the interaction between *C. neoformans* and the host cell.

Our group had described extracellular proteins in *Paracoccidiodes.* Proteins lacking predicted signal peptides, such as enolase, have been shown to be secreted by *Paracoccidioides* into the extracellular medium [22]. Additionally, formamidase activity has been detected in *Paracoccidiodes* cell-free extracts [23]. An aspartyl protease has been reported in *Paracoccidioides* culture supernatants [24], and a serine protease, which depicted increased levels of transcript during nitrogen starvation, has also been identified in *Paracoccidioides* culture supernatants, thereby indicating the potential function of this protein in fungus nitrogen acquisition [25]. The *Paracoccidioides* serine protease transcript is induced in yeast cells infecting murine macrophages [25] and during the incubation of yeast cells with human plasma [26], thereby suggesting that the protein plays a putative role in *Paracoccidioides* interaction with the host cell.

In a recent study, it has been shown that extracellular vesicles in *Paracoccidioides* yeast cells, *Pb*18, carry antigenic molecules (α-galactopyranosyl epitopes) that are recognized by the sera of PCM patients [20]. The vesicle and vesicle-free fractions were used to identify the extracellular proteome via LC/MS [27]. Eighty-five proteins were identified exclusively in the vesicle fractions compared with 140 proteins that were detected solely in the vesicle-free fractions, for which 120 sequences displayed overlap in both fractions. The authors described 75 extracellular proteins that were common to *Paracoccidiodes, Pb*18, and at least two of the other analyzed fungal species.

In the current study, we identified the most abundant constituents of the extracellular proteome in mycelia and *Paracoccidioides*, *Pb*01, yeast cells. We also identified proteins differentially secreted by mycelia and yeast cells and investigated the influence of protein export inhibition on the phagocytic ability of macrophages in an attempt to further understand the role that extracellular proteins play in the establishment and pathogenesis of the PCM.

## Materials and Methods

### 2.1. Microorganism and culturing conditions

*P. lutzii*, *Pb*01, (ATCC MYA-826) was used in the experiments conducted in this study. The cells were cultured in Fava Netto's medium [0.3% (w/v) proteose peptone, 1% (w/v) peptone, 0.5% (w/v) meat extract, 0.5% (w/v) yeast extract, 4% (w/v) glucose, and 0.5% (w/v) NaCl, pH 7.2] at 36 C and 22°C, for yeast cells and the mycelia phase, respectively. Yeast cell viability and growth were evaluated in triplicate every 6 hours. A trypan blue dye exclusion test was performed to ensure over 95% cell viability during the incubation process.

### 2.2. Preparation of extracellular protein extracts

The yeast and mycelia extracellular proteins were prepared by inoculating 50 µg/mL of wet weight cells in Fava Netto's liquid medium, and the cells were maintained while shaking (200 rpm) for 24 hours at 36°C and 22°C, respectively. After incubation, the cells were removed by centrifugation at 10,000 g for 30 min at 4°C. The culture supernatant was sequentially filtered through 0.45 µm-pore and 0.22 µm-pore membrane filters. Culture filtrates were concentrated and subsequently washed three times with ultrapure water via ultracentrifugation through a 10-kDa molecular weight cut off in ultracel regenerated membrane (Amicon Ultra centrifugal filter, Millipore, Bedford, MA, USA). The protein concentrations were determined via Bradford assay [28]. For proteomic analysis, three biological replicates of protein samples obtained from three different experiments were performed for yeast cells and mycelia. The three yeast and mycelia cell-free supernatant samples were assessed for the presence of *Paracoccidioides* DNA via PCR, which if positive indicated fungal cellular lyses in the samples as described below.

### 2.3. The Polymerase Chain Reaction (PCR) analysis

The genomic DNA isolated from *Paracoccidiodes* mycelia and yeast cells was obtained according to standard protocol [29]. The PCR reactions were performed with cell-free supernatant (2 µL) and genomic DNA samples as follow: 40 cycles of 94°C for 30 s, 55°C for 30 s, and 72°C for 1 min. A 1622-bp PCR product was generated using sense S2 (5′- ATGGGTCTCAAGGGAATTC-3′) and antisense At2 (5′-CATCCCCTACTTCATTC-3′) oligonucleotides for the gene encoding formamidase (GenBank accession number AY163575). The PCR amplicons were detected via 1% (w/v) agarose gel electrophoresis with ethidium bromide staining. PCR sensitivity was assessed using *Paracoccidioides Pb*01 genomic DNA (at five dilutions) as a template (50 ng to 1 pg).

### 2.4. Two-dimensional gel electrophoresis (2-DE)

For the first dimension, each sample containing 500 µg of total protein was treated with a 2D-Clean-up Kit (GE Healthcare, Uppsala, Sweden) according to the manufacturer's instructions. The amount of protein (500 µg) was diluted to 200 µL of the final sample volume and corresponded to 30 g of wet weight cells (Section 2.2). The proteins samples were diluted in 250 μL of rehydration solution containing 7 M urea, 2 M thiourea, 2% (w/v) 3-[(3-Cholamidopropyl) dimethylammonio]-1-propanesulfonate (CHAPS), 0.002% (w/v) dithiothreitol (DTT), 0.5% (v/v) ampholyte 3–11 and trace amounts of bromophenol blue [30]. These samples were loaded onto a 13 cm Immobiline™ DryStrip gel with a pH linear range of 3–11 in a Multiphor-II Electrophoresis System (GE Healthcare, Uppsala, Sweden). The samples were separated according to their isoelectric points at 20°C with a current of 50 µA/strip. The following program was applied: 30 V for 14 hours; 500 V for 500 Vh (step); 1 kV for 800 Vh (gradient); 8 kV for 11.3 kVh (gradient) and 8 kV for 2.9 kVh (step). After isoelectric focusing, the strips were equilibrated twice for 40 min in equilibration buffer [50 mM Tris-HCl pH 8.8, 6 M urea, 30% (v/v) glycerol, 2% (w/v) SDS and 0.002% (w/v) bromophenol blue] containing 18 mM DTT and 135 mM iodoacetamide [31]. The second dimension (SDS-PAGE) was performed with 12% polyacrylamide gels using a vertical system (GE Healthcare) and standard Tris/glycine/SDS buffer for one hour at 150 V and 250 V until the end of the run at 12°C. The gels were stained with Coomassie brilliant blue (PlusOne Coomassie Tablets PhastGel Blue R-350, GE Healthcare) according to the manufacturer's instructions.

### 2.5. Image analysis

The 2-D gel images were obtained using an Image Scanner III (GE Healthcare). 2D gel spot detection, matching and intensity calculations were performed with Image Master 2D Platinum v7.0 (GE Healthcare). Three independent samples were prepared for each fungal phase to ensure reproducibility. The final set contained six gel images. Software matching between the images was performed, and the matching process was thoroughly assessed via visual inspection. The volume percentage of the spots was used for statistical calculations and the determination of overexpressed proteins.

### 2.6. 2D-gel statistical analysis

To compare the differences in protein expression between *Paracoccidioides* mycelia and yeast cells the ANOVA test was applied considering statistically significant p-value ≤0.05. To compare the proteins with multiple isoforms, the sum of the percentage of the volumes (in relation to the total proteins) of each isoform was first obtained in triplicate. Next, the sum of the percentage of volumes for the proteins was used for statistical analysis, which was performed to determine the significant differences in expression profiles between *Paracoccidioides* mycelia and yeast proteins. All statistical calculations were performed using the software STATISTICA version 7.0. (Statsoft Inc., 2005). The spectrometry analyses were performed on spots displaying significant alterations (≥ fold change of 2.5) between yeast and mycelia samples and spots displaying similar expression levels (common spots).

### 2.7. Mass spectrometry analysis

Spots of interest were manually excised and digested as previously described [32], [33]. Briefly, the gel pieces were resuspended in 100 μL acetonitrile (ACN) and dried in a speed vacuum. The gel pieces were then reduced with 10 mM DTT and alkylated with 55 mM iodoacetamide. The supernatant was then removed, and the gels were washed with 100 μL ammonium bicarbonate by vortexing for 10 min. The supernatant was removed, and the gel pieces were dehydrated in 100 μL of a solution containing 25 mM ammonium bicarbonate/50% (v/v) ACN, vortexed for 5 min, and centrifuged. This step was then repeated once. Next, the gel pieces were dried in a speed vacuum and 12.5 ng/mL trypsin (sequencing grade modified trypsin, Promega, Madison, WI, USA) solution was added followed by a rehydration step performed on ice at 4°C for 10 min. The supernatant was removed, 25 μL of 25 mM ammonium bicarbonate was added and the supernatant was then incubated at 37°C for 16 hours. Following digestion, the supernatant was placed in a clean tube. Next, 50 μL 50% (v/v) ACN and 5% (v/v) trifluoroacetic acid (TFA) were then added to the gel pieces. The samples were vortexed for 30 min, sonicated for 5 min, and the solution was then combined with the aqueous extraction above. The samples were dried in a speed vacuum, the peptides were solubilized in 10 µL ultrapure water, and the samples were subsequently purified in ZipTip® Pipette Tips (ZipTips® C18 Pipette Tips, Millipore, Bedford, MA, USA). Two microliters of each peptide sample were deposited onto a matrix-assisted laser desorption ionization quadrupole time-of-flight mass spectrometry (MALDI-Q-TOF MS) target plate and dried at room temperature. Next, the peptide mixture was covered with 2 μL of matrix solution (10 mg/ml α-cyano-4-hydroxyciannamic acid matrix in 50% (v/v) ACN and 5% (v/v) trifluoroacetic acid). The mass spectra were recorded in the positive reflectron mode on a MALDI-Q-TOF mass spectrometer (SYNAPT, Waters Corporation, Manchester, UK).

The search against the NCBI non-redundant database using the MS/MS data was performed using Mascot software v. 2.4 (http://www.matrixscience.com) (Matrix Science, Boston, USA). The Mascot MS/MS ion search parameters were as follows: tryptic peptides with one missed cleavage allowed; fungi taxonomic restrictions; fixed modifications: carbamidomethylation of Cys residues; variable modifications: oxidation of methionine; and an MS/MS tolerance of 0.6 Da. The identified proteins were described in functional categories according to the MIPS Functional Catalogue Database (http://fsd.riceblast.snu.ac.kr).

For the identification of the MS spectra using the NCBI database, we included the analysis of post-translational modifications (PTMs) for multiple identified proteins/isoforms. We included variable modifications in the search as follows: the acetylation of lysine and the phosphorylation of serine/tyrosine/tryptophan. All proteins/isoforms that presented matches with predicted modified peptides were selected for manual spectral analysis.

### 2.8. *In silico* analyses

A number of programs available online were used for the characterization of the identified extracellular proteins. The identified proteins were analyzed using SignalP 3.0 software (http://www.cbs.dtu.dk/services/SignalP/) for the prediction of signal peptides. For cases when the signal sequence was detected, the protein was considered to be secreted via a classical pathway. For the prediction of secreted proteins by non-classical pathways, the software SecretomeP 2.0 (http://www.cbs.dtu.dk/services/SecretomeP/) was employed. Using the prediction methods, a score ranging between 0 and 1 was assigned to each protein in which a score equal or higher than 0.5 was considered indicative of secretion.

The Fungal Secretome Database (FSD) (http://fsd.riceblast.snu.ac.kr) was used to analyze our results. The FSD provides a summary of putative secretory proteins by *in silico* analysis based on prediction programs for 158 fungal/oomycete genomes, including *Paracoccidioides Pb*01 [34]. Each protein sequence identified in this study was also analyzed for adhesin function using the Faapred web server (http://bioinfo.icgeb.res.in/faap/query.html) and for GPI anchor properties using big-PI Fungal Predictor software (http://mendel.imp.univie.ac.at/gpi/fungi/gpi_fungi.htm). The Faapred web server allows for the prediction of adhesins in the fungal proteomes using an accurate method with scores higher than or equal to 0.8 [35]. The big-PI Fungal Predictor is a sensitive prediction tool used for the recognition of C-terminal motifs capable of GPI lipid anchoring in fungal sequences [36].

The ORF sequences of the identified proteins of *Paracoccidioides Pb*01 yeast cells were submitted for Blast analysis using the NCBI website (http://www.ncbi.nlm.nih.gov/) against orthologues previously reported for *Paracoccidioides Pb*18 [27] as well the secretomes of the pathogenic fungi *Histoplasma capsulatum* [10], [18], *Cryptococcus neoformans* [19] and *Aspergillus fumigatus* [37].

### 2.9. Enzymatic activity assay

To determine the activity of formamidase (FMD), ammonia formation was assessed as previously described [23]. Protein extracts were obtained as described in Section 2.2. Next, 1 μg of total protein extract was added to 200 μL of 100 mM formamide substrate solution in 100 mM phosphate buffer containing 10 mM EDTA, pH 7.4. The samples were incubated at 37°C for 30 min. Next, 400 μL phenol-nitroprusside and 400 μL alkaline hypochlorite were added to the samples. The samples were then incubated for 6 min at 50°C, and the absorbance was measured at 625 nm. The amount of ammonia released for each sample was determined using a standard curve. One unit (U) of FMD activity was defined as the amount of enzyme required to hydrolyze 1 μmol formamide per mg total protein per minute (µmol/mg/min). The levels of superoxide dismutase (SOD) and glutathione S-transferase (GST) activity were measured using commercially available kits (Superoxide dismutase Assay Kit and Glutathione-S-Transferase (GST) Assay Kit, Sigma-Aldrich, Co., St. Louis, MO, respectively). The SOD Assay Kit includes Dojindo's highly water-soluble tetrazolium salt (WST-1), which produces a water-soluble formazan (WST-1 formazan) dye upon reduction with a superoxide anion. The reaction product (WST-1 formazan) absorbs at 440 nm, and it is proportional to the amount of superoxide anion. The levels of SOD activity, which were defined as inhibition rate %, were quantified by measuring the decrease in the color development at 440 nm. The GST Assay Kit employs 1-Chloro-2,4-dinitrobenzene (CDNB) to produce GS-DNB by conjugation of the thiol group of glutathione (GSH). The reaction product (GS-DNB) absorbs at 340 nm, and the rate of increase in the absorption is directly proportional to the GST activity of the sample. The GST-specific activity is defined as µmol of GS-DNB per mg of total protein per minute (µmol/mg/min). The enzymatic activity results represent the mean of three independent determinations, and statistical comparisons were performed using the Student's t test. The samples with *p*-values ≤0.05 were considered statistically significant.

### 2.10. Brefeldin A treatments and *Paracoccidioides* internalization by macrophages

Brefeldin A (BFA, Sigma), was used to evaluate the role of conventional protein secretion on *Paracoccidioides* internalization by macrophages. Stock solutions (1 mg/mL) of Brefeldin A (BFA) were prepared in methanol and stored at −20°C until use. The yeast cells were cultivated in Fava Netto's liquid medium in either the absence or presence of BFA (6 μg/mL) for 6, 12 and 24 hours. The cell-free supernatant samples were obtained as described above, reduced to an equal final volume (1 mL), and processed for one-dimensional electrophoresis (SDS-PAGE). Next, 30 μL of each cell-free supernatant sample was loaded onto a 12% SDS PAGE gel, and the proteins were separated via electrophoresis. The gels were run at 150 V for approximately 2 hours and visualized using Coomassie brilliant blue staining.

Bone marrow-derived macrophages (BMM) were obtained as previously described from 6 to 8-week-old BALB/c male mice [38]. The macrophages were generated by extracting bone marrow cells from the femurs of BALB/c male mice, and the cell cultures were incubated at 37°C with 5% CO_2_ in RPMI medium (RPMI 1640, Vitrocell, Brazil) supplemented with 10% (v/v) fetal bovine serum and GM-CSF (Recombinant Murine Granulocyte Macrophage Colony Stimulating Factor, PeproTech-Brasil FUNPEC, Brazil) at 10 ng/mL for 8–10 days, which prompts differentiated macrophages to adhere to the plastic-bottom plates.

To determine the number of adhered/internalized fungi cells by BMM, the differentiated macrophages were quantified and plated at 5×10^5^ cells per well on glass coverslips in 6-well culture plates and infected with *Paracoccidioides* yeast cells at a 1:5 ratio macrophage: yeast. The cells were co-cultivated for 12 hours at 37°C in 5% CO_2_ to allow for fungi adhesion and/or internalization. The supernatants were then aspirated, the monolayer was gently washed twice with PBS 1X to remove any non-adhered/internalized yeast cells, and the samples were processed for microscopy. The glass coverslips were fixed with methanol and stained with Giemsa (Sigma). A total of 200 macrophages were quantified to determine the average number of adhered/internalized fungal cells [39].

The number of viable fungi after co-cultivation with macrophages was determined by quantifying the number of colony forming units (CFUs). The cells were rinsed twice with PBS 1X to remove any non-internalized yeast cells. The wells were washed with distilled water to promote macrophages lysis, and the suspensions were collected in individual tubes. Fifty microliters of cell homogenate was plated in BHI medium supplemented with 5% (v/v) fetal bovine serum and incubated at 37°C in 5% CO_2_ for 15 days_._ The number of CFUs was expressed as the mean value ± the standard deviation. A portion of this macrophage homogenate was processed for immunoblot analysis. The experiments were performed in triplicate, and the statistical analyses were performed using the Student's t test.

BFA treatment was performed to evaluate the role of extracellular proteins in the adhesion/internalization of *Paracoccidioides* by macrophages. *Paracoccidioides* yeast cells were cultivated in Fava Netto's liquid medium in either the absence (control) or presence of BFA (6 μg/mL) for 24 hours prior to the infection of macrophages. The effect of Brefeldin on macrophages was evaluated by adding BFA posteriorly to the co-culture of macrophages with fungal cells (not in the preliminary culture of *Paracoccidioides* cells) as an additional control (data not show). To test the biological activity of the secreted proteins released by *Paracoccidioides* yeast cells, we evaluated whether the addition of concentrated culture supernatant influenced the adhesion/internalization of *Paracoccidioides* by macrophages. We added 30 µL of extracellular protein extract of *Paracoccidioides* yeast cells (2 µg/µL) to the macrophage and fungal cell co-culture. The number of adhered/internalized yeast cells by BMM and the survival rate (viable fungi after co-cultivation) were determined. The results were compared with the control (absence of extracellular proteins and BFA). All animal work was conducted in accordance with the international rules for animal experimentation. The animal protocol was approved by the Universidade Federal de Goiás ethical committee of animal treatment (Number: 192/2011).

### 2.11. Sample preparation and nanoUPLC-MS^E^ acquisition

*In vitro* cultured macrophages were infected with *Paracoccidioides Pb*01 yeast cells, and co-cultivated for 12 hours at 37°C in 5% CO_2._ The infected and non-infected macrophages (BMM) were incubated in distilled water to promote macrophages lysis and centrifuged at 10,000× *g* for 5 min to separate the macrophage cytosol from the pellet containing host nuclei, cytoskeleton, and *Paracoccidioides* yeast cells. The macrophage lysate supernatant was sequentially filtered through a 0.22 µm-pore-size membrane filter, washed three times with ultrapure water by ultracentrifugation using a 10-kDa molecular weight cut off in ultracel regenerated membrane (Amicon Ultra centrifugal filter, Millipore, Bedford, MA, USA), and concentrated to a final volume of 1 mL. The protein concentrations were determined via Bradford assay [28].

To analyze the expression profile of the *Paracoccidioides* secreted proteins with *in vitro* cultured macrophages, the samples (obtained as described above) were analyzed using nanoscale liquid chromatography coupled with tandem mass spectrometry. Sample aliquots (100 µg) were prepared for nanoLC-MS/MS as previously described [40]. The digested peptides were further separated via nanoUPLC-MS^E^ and analyzed using a nanoACQUITY^TM^ system (Waters Corporation, Manchester, UK). The MS data obtained via UPLC-MS were processed and examined using the ProteinLynx Global Server (PLGS) version 2.4 (Waters Corporation, Manchester, UK). For protein identification and quantification level analysis, the observed intensity measurements were normalized with the identified peptides of the digested internal standard.

### 2.12. Immunoblot analysis of proteins secreted by *Paracoccidioides* in macrophages

For immunoblot analysis, the same samples used for nanoUPLC mass spectrometry analysis were probed using triosephosphate isomerase and enolase antibodies [22], [41]. Thirty micrograms of protein sample was loaded onto a 12% SDS-PAGE gel and separated by electrophoresis. The gels were run at 150 V for approximately 2 hours. The proteins were transferred from the gels to nitrocellulose membranes at 30 V for 16 h in a buffer containing 25 mM Tris-HCl (pH 8.8), 190 mM glycine and 20% (v/v) methanol. The gels were stained with Ponceau red to verify complete protein transfer. Next, each membrane was incubated in blocking buffer (1X PBS, 1.4 mM KH2PO4, 8 mM Na2HPO4, 140 mM NaCl, 2.7 mM KCl (pH 7.3), 5% (w/v) nonfat dried milk, and 0.1% (v/v) Tween 20) for 2 h. The membranes were washed with buffer (1X PBS and 0.1% (v/v) Tween 20) and incubated with primary antibodies for 2 h at room temperature. The primary antibodies were used at a dilution of 1 antibody/40000 buffer (v/v). The primary polyclonal antibody employed was either anti-enolase [22] or anti-triosephosphate isomerase [41]. The membranes were then washed in blocking buffer three ×15 min. The membranes were incubated with the appropriate conjugated secondary antibody, either a horseradish-peroxidase conjugated anti-rabbit or anti-mouse IgG, at a 1/5000 (v/v) ratio, and the blots were developed using an enhanced chemioluminescence detection system (ECL, GE Healthcare).

## Results

### 3.1. Validation of the extracellular protein extraction method

**Figure 1:**
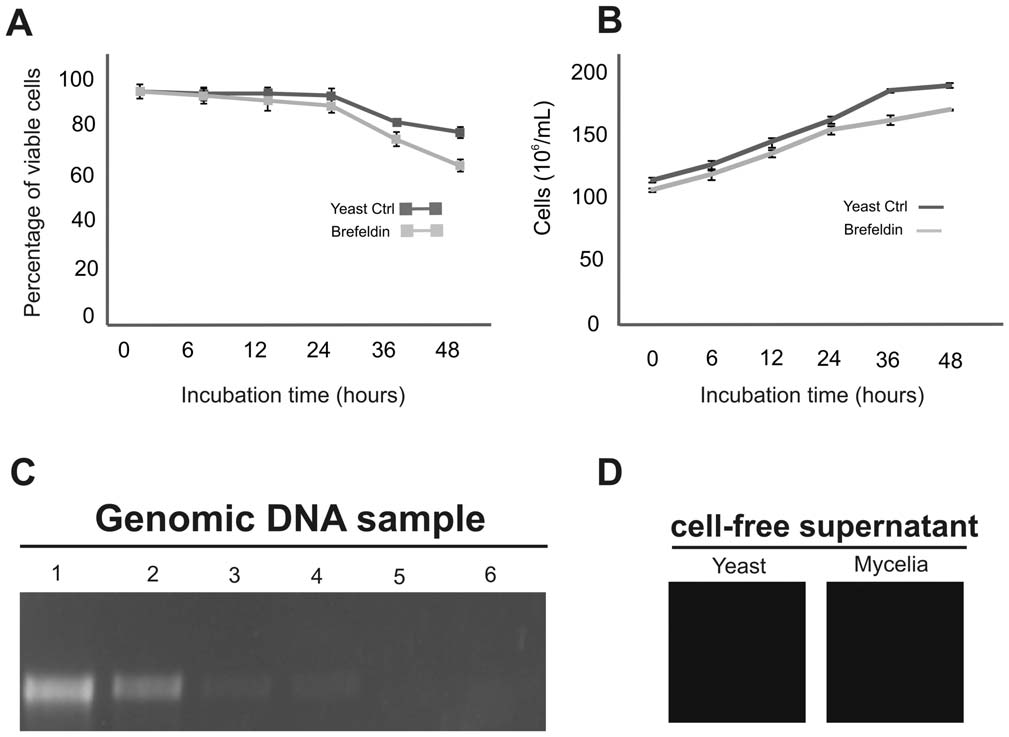
Validation of the extracellular protein extraction method. **A-** The viability of *Paracoccidioides* yeast cells incubated in Fava Netto's liquid medium (dark gray square) and the incubation of yeast cells in Fava Netto's liquid medium containing 6 µg/mL Brefeldin A (light gray square). Viability was assessed using trypan blue staining. The error bars represent the standard deviation of three biological replicates. **B-** The growth of *Paracoccidioides* yeast cells in liquid medium in either the absence (dark line) or presence of 6 µg/mL Brefeldin A (light gray line). Culture growth was evaluated by quantifying the number of yeast cells per mL. The error bars represent the standard deviation of three biological replicates. **C-** PCR sensitivity for the formamidase gene was assessed using *Paracoccidiodes Pb01* genomic DNA (at five dilutions) as a template (50 ng to 1 pg). Lanes: 1 −50 ng; 2 −5 ng; 3 −50 pg; 4 −5 pg; 5 −1 pg; 6 - negative control (without genomic DNA). The formamidase PCR amplicons were assessed via 1% agarose gel electrophoresis and stained with ethidium bromide. **D-** The yeast and mycelia cell-free supernatant samples (2 µL) were assessed for the presence of *Paracoccidiodes* DNA via PCR using oligonucleotides specific for the formamidase gene.

Viability analysis was performed with the yeast cells. Using trypan blue staining, we observed yeast cell viability over 95% during a 24-h cultivation in liquid medium (Figure 1A). To standardize sample acquiring, PCR analysis was performed to assess for the *Paracoccidioides* formamidase gene. The sensitivity of the PCR assay was assessed using FMD specific primers with a serial dilution of the genomic DNA sample (ranging from 50 ng to 1 pg). The smallest quantity of DNA that was amplified by PCR was 5 pg (Figure 1C, lane 4). This result implies that technique is able to amplify the FMD gene with only 5 pg DNA present in the cell-free supernatant. As shown in Figure 1D, the FMD gene was not detected in the yeast and mycelia cell-free supernatants. These data both contribute to the hypothesis that the cell lyses failed to influence the extracellular protein profiles of the extracts obtained for proteomic analysis and validate the extraction method of extracellular proteins expressed by *Paracoccidioides*. Additionally, none of the secreted had motifs for GPI-anchor (data not shown), as evidenced by *in silico*, suggesting that the presence of the proteins in the secretome was not due to the accidental release from the fungal surface.

### 3.2. Analysis of secreted proteins in *Paracoccidioides Pb*01 yeast cells and mycelia

**Figure 2:**
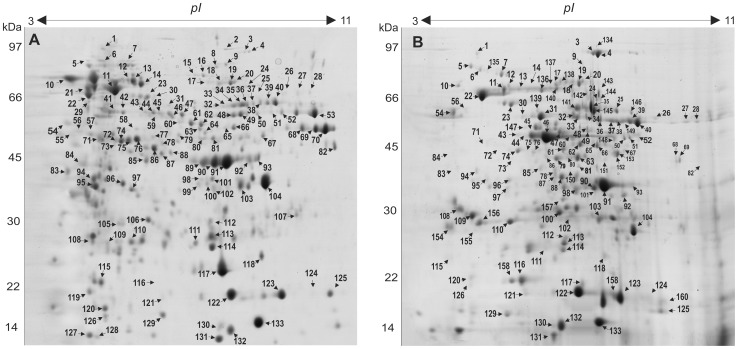
Proteins detected in the secretome of yeast cells and mycelia of *Paracoccidiodes* via 2D-gel analysis. Protein profile generated after the separation of the secreted fraction of proteins by yeast cells (**A**) and mycelia (**B**) using 2D-eletrophoresis (first dimension: IEF pH range 3 –11 non-linear, second dimension: 12% (w/v) SDS-PAGE) and visualized using Coomassie brilliant blue staining. The 2-D gel images of three biological replications of each phase were compared to identify the differential expression levels of proteins using Image master 2D Platinum software. The protein spots that were identified via MS/MS are numbered and listed in Table S1. The pH gradient is shown above the gel, and the molecular mass protein standards (kDa) are indicated to the left of the gels.

In this study, we applied a proteomic strategy to identify proteins constitutively secreted by *Paracoccidioides Pb*01 and analyzed the proteins preferentially secreted by yeast cells and mycelia. Figure 2 depicts the representative two-dimensional gel of both phases performed in biological triplicates. Proteins were distributed in a molecular mass range from 14.10 to 105.25 kDa and an experimental *pI* value ranging from 3.76 to 10.18 was observed. The figure displays the presence of constitutive and differentially expressed proteins in both secretomes.

**Figure 3:**
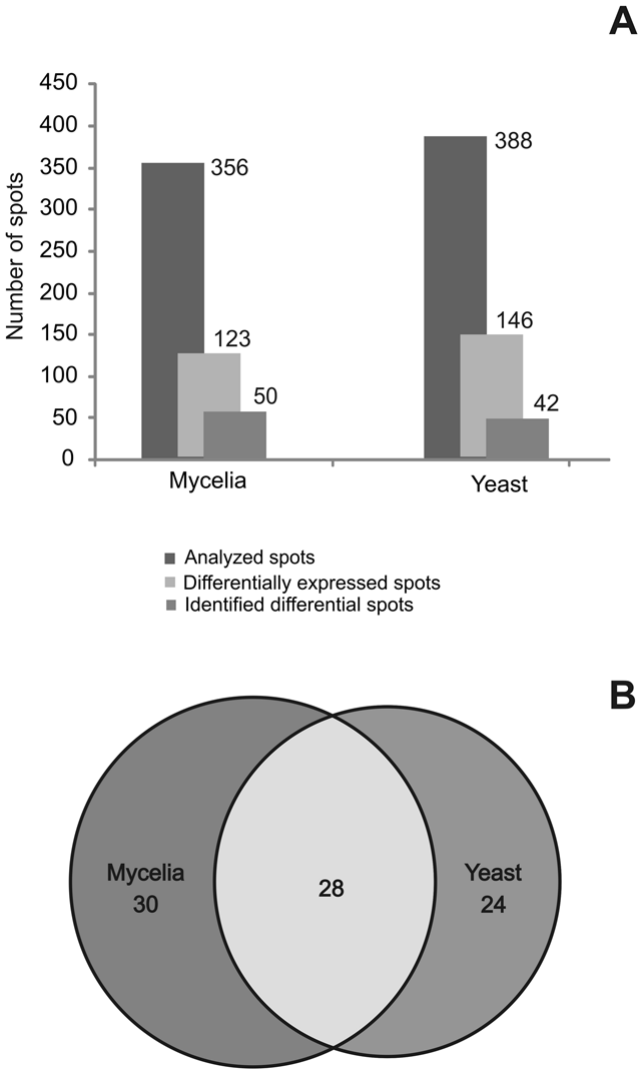
Graphic summation of proteomics analysis. **A-** The comparative analysis using Image master 2D Platinum software displays the analyzed spots and the preferentially expressed spots in mycelia and yeast secretomes, while the mass spectrometry analyses displays the identified differentially expressed spots. **B-** The Venn diagram shows the number of identified proteins via MS/MS. The preferentially secreted extracellular proteins include those with statistically significant alteration in the mycelia and yeast secretome, while the constitutive proteins refer to those with similar expression patterns.

Figure 3 depicts the graphic summation of the proteomic analysis, which revealed an average of 356 spots for the mycelia and 388 spots for the yeast secretome, which were matched and assessed for statistical significance using an ANOVA test to compare the differences in protein expression between the two fungal phases. After performing the statistical analysis, it was determined that 123 and 146 spots (i.e., proteins) were differentially secreted by mycelia and yeast cells, respectively (Figure 3A).

Coomassie G-250 Blue Stained maps were used for MS identification of the individual protein spots. A total of 160 proteins/spots were identified unambiguously (displayed in Table S1), which displays all proteins and isoforms identified in the secretome analysis. The identified proteins/isoforms were analyzed using several prediction programs. While not all secreted proteins/isoforms have canonical signal peptides, 20 of the 160 (12.5%) contained a potential N-terminal secretion signal (Mean S score ≥0.5, Table S1). The proteins/isoforms that presented the signal peptide consensus included enzymes such as glycosyl hydrolase, beta glucosidase, enoyl-CoA hydratase, aminopeptidase and disulfide isomerase. Using the Secretome P algorithm, 84 of 160 (52.5%) proteins/isoforms were predicted to be secreted molecules, including aconitase, enolase, fructose-biphosphate aldolase, 2-methylcitrate synthase, glyceraldehyde-3-phosphate dehydrogenase, dipeptidyl-peptidase, thioredoxin-like proteins and peroxisomal catalase.

Regarding the proteins identified in the secretomes, fungal adhesin prediction analysis using the Faapred web server revealed that approximately 21% of the *Paracoccidioides* secretome (33 proteins/isoforms) was predicted to be adhesin-like proteins, such as enolase, glyceraldehyde-3-phosphate dehydrogenase, and triosephosphate isomerase (Table S1). All identified proteins/isoforms were grouped into Enzyme Classification (EC) according to guidelines of the Nomenclature Committee of the International Union of Biochemistry and Molecular Biology (NC-IUBMB) as listed in Table S1. More than half of the *Paracoccidioides Pb*01 secretome comprises enzyme-like proteins (64%), and the most prevalent enzyme classes found were oxidoreductases (17%), transferases (17%), and hydrolases (14%) as depicted in Figure S1.

### 3.3. Constitutive proteins in the secretome of *Paracoccidioides Pb*01

In this report, we described the proteins constitutively present in the secretome of *Paracoccidioides Pb*01 mycelia and yeast cells. For this analysis, the isoforms of each protein were combined to perform the statistical analysis. Twenty eight proteins were constitutive to mycelia and yeast secretomes (Figure 3B). Most of these proteins were grouped into the functional category of metabolism, energy and protein fate. The enzymes in the energy category, including enolase, fructose-bifophosphate aldolase, glyceraldehyde-3-phosphate dehydrogenase and phosphogycerate kinase, were abundant in both secretomes as depicted in Table 1.

**Table 1:** Constitutive proteins secreted by *Paracoccidioides Pb*01 yeast and mycelia.

General Information number (NCBI) 1	Protein description	Number of isoforms in *Paracoccidioides* secretome 2	Amount of isoform abundances 3	ANOVA (*p*-value) 4	SignalP Score ≥0.5 5	SecretomeP Score ≥0.5 6
**1. CELL RESCUE, DEFENSE and VIRULENCE**						
gi|295659787	heat shock protein Hsp88	1	1.24	0.3645	NO	NO
gi|295659837	heat shock protein SSB1	1	2.77	0.0779	NO	0.8618
gi|295669402	Mn superoxide dismutase	1	1.82	0.3780	NO	0.8823
**2. METABOLISM**						
**2.1. Amino Acid Metabolism**						
gi|295667902	aminomethyltransferase	1	1.52	0.2302	NO	0.6367
gi|295658698	fumarylacetoacetase	4	1.50	0.7047	NO	0.7492
gi|295658947	O-acetylhomoserine (thiol)-lyase	1	1.00	0.9268	NO	0.7930
gi|225683737	spermidine synthase	1	1.93	0.0864	NO	NO
**2.2. Nucleotide Metabolism**						
gi|295674697	adenosine kinase	2	0.21	0.0646	NO	NO
**2.3. Secundary Metabolism**						
gi|295666938	nucleoside diphosphate kinase	1	1.01	0.5725	NO	NO
**2.4. Phosphate Metabolism**						
gi|295662360	mannitol-1-phosphate 5-dehydrogenase	3	0.42	0.0766	NO	NO
**2.5. C-Compound and Carbohydrate Metabolism**						
gi|295667790	beta-glucosidase	1	1.63	0.2116	0.999	0.8279
gi|295663469	glycosyl hydrolase	1	1.77	0.1350	0.996	0.8631
gi|295665168	TOS1	1	1.00	0.7772	0.999	0.9249
**3. ENERGY**						
**3.1. Glycolysis and Gluconeogenesis**						
gi|295672732	enolase	4	0.67	0.0832	NO	0.5000
gi|295671120	fructose-bisphosphate aldolase	5	398.84	0.0549	NO	0.6628
gi|295658119	glyceraldehyde-3-phosphate dehydrogenase	2	0.76	0.4655	NO	0.9120
gi|295669690	phosphoglycerate kinase	1	1.05	0.3639	NO	0.6626
gi|225678203	NmrA-like family protein	1	1.01	0.5624	NO	0.5898
**3.3. Tricarboxylic-acid Pathway**						
gi|295673937	malate dehydrogenase	1	1.36	0.0834	0.764	0.8163
gi|295668473	dihydrolipoyl dehydrogenase	3	17.92	0.0541	0.571	NO
**4. CELL CYCLE AND DNA PROCESSING**						
gi|295664474	cell division cycle protein	1	1.24	0.2116	NO	NO
gi|295658863	Cofilin/tropomyosin-type actin- binding family protein	2	1.91	0.0093	NO	NO
gi|295672736	DNA damage checkpoint protein rad24	2	0.86	0.0693	NO	NO
**5. PROTEIN FATE (folding, modification, destination)**						
gi|295663907	peptidyl-prolyl cis-trans isomerase A2	1	1.06	0.4941	0.975	0.8963
gi|295672668	peptidyl-prolyl cis-trans isomerase B	2	191.86	0.0920	0.928	NO
gi|295662699	peptidyl-prolyl cis-trans isomerase cypE	1	1.12	0.2723	NO	0.8712
gi|295672447	peptidyl-prolyl cis-trans isomerase H	3	144.1	0.0755	NO	0.7266
gi|295665666	Grp1p protein	1	1.06	0.6021	NO	0.9061
**6. UNCLASSIFIED PROTEINS**						
gi|295667926	conserved protein	1	1.01	0.7060	0.972	0.9280
gi|295657286	conserved protein	1	1.00	0.7950	NO	0.8050

1 NCBI database general information number (http://www.ncbi.nlm.nih.gov/).

2 Number of identified isoforms of protein in *Paracoccidioides, Pb01* secretome.

3 The average of amount of values of abundances of all identified isoforms used to statistical test.

4 ANOVA – statistically significant differences are considered with *p*<0.05 (*).

5 Secretion prediction according to Signal P 3.0 server, the number corresponds to signal peptide probability (http://www.cbs.dtu.dk/services/SignalP/).

6 Secretion prediction according to Secretome P 2.0 server, the number corresponds to neural network that exceeded a value of 0.5 (NN-score **≥**0.50) (http://www.cbs.dtu.dk/services/SecretomeP/).

### 3.4. Extracellular proteins upregulated in *Paracoccidioides Pb*01 mycelia

A comparative analysis was performed between the extracellular protein profiles of mycelia and yeast cells. Based on protein categorization, 30 proteins were preferentially secreted by mycelia compared with yeast cells (Figure 3B and Table 2). The proteins identified are implicated in a variety of biological processes, such as cell rescue, cell defense, cell virulence, metabolism, energy, cell cycle, DNA processing, protein fate and protein synthesis (Table 2). Proteins that play a role in cell rescue, defense and virulence include the heat shock protein SSC1, disulfide isomerase, glutathione reductase, peroxisomal catalase, thioredoxin reductase and the TCTP family of proteins. Enzymes involved in the metabolism of amino acids were abundant in the mycelia secretome. Some of the proteins expressed in the mycelia secretome were significantly upregulated compared with the yeast cells, including the Cofilin/tropomyosin-type actin-binding family of proteins (fold change of 1911.74), peroxisomal catalase (fold change of 61.29), glutamate carboxypeptidase (fold change of 51.03), aconitase (fold change of 56.98) and heat shock protein SSC1 (fold change of 35.47) (Table 2). Interestingly, several proteins were detected exclusively in the mycelia secretome, including detoxification proteins such as thioredoxin reductase and disulfide-isomerase tigA, proteins that play a role in the metabolism of amino acids, and proteins involved in energy production. The proteins expressed exclusively in the secretome of mycelia (compared with the yeast phase) are summarized in Table S2.

**Table 2:** Preferentially secreted proteins by *Paracoccidioides Pb*01 mycelia.

General Information number (NCBI) 1	Protein description	Number of isoforms in mycelia 2	Amount of isoform abundances 3	ratio of M/Y abundance 4	ANOVA (*p*-value) 5	SignalP^9 ^Score ≥0.5 6	SecretomeP Score ≥0.5 7
**1. CELL RESCUE, DEFENSE and VIRULENCE**							
gi|295671569	heat shock protein SSC1	6	8.87	35.47	0.0171*	NO	0.6791
gi|295670457	disulfide-isomerase tigA	1	0.07		**	NO	NO
gi|295664022	glutathione reductase	4	0.87	4.98	0.0121*	NO	NO
gi|225681400	peroxisomal catalase	2	0.28	61.29	0.0181*	NO	0.9389
gi|295661107	thioredoxin reductase	1	1.47		**	NO	0.966
gi|295656848	TCTP family protein	1	1.08		**	NO	NO
**2. METABOLISM**							
**2.1. Amino Acid Metabolism**							
gi|295671621	choline dehydrogenase	1	0.09		**	NO	NO
gi|295659538	Cobalamin-independent methionine synthase	1	0.04		**	NO	NO
gi|295664250	histidine biosynthesis trifunctional protein	1	0.32		**	NO	0.6345
gi|295661139	methylmalonate-semialdehyde dehydrogenase	2	0.41		**	NO	0.8078
gi|295659992	serine hydroxymethyltransferase	1	0,15		**	NO	NO
**2.2. Nucleotide Metabolism**							
gi|295668873	phosphoribosylamine-glycine ligase	1	0.14		**	NO	NO
**2.3. C-Compound and Carbohydrate Metabolism**							
gi|295665123	aldehyde dehydrogenase	1	0.03		**	NO	NO
**2.4. Nitrogen metabolism**							
gi|295668479	formamidase	5	1.60	4.59	0.0033*	NO	NO
**3. ENERGY**							
**3.1. Glycolysis and Gluconeogenesis**							
gi|295659988	2,3-bisphosphoglycerate -independent phosphoglycerate mutase	1	1.20		**	NO	0.6367
gi|295670663	triosephosphate isomerase	1	1.52	2.52	0,0132*	NO	NO
**3.2. Oxidation of fatty acids**							
gi|295659859	acetyl-CoA acetyltransferase	2	0.29	6.47	0.0477*	NO	0.8393
gi|295670934	electron transfer flavoprotein-ubiquinone oxidoreductase	1	0.10		**	NO	0.8751
**3.3. Tricarboxylic-acid Pathway**							
gi|295664721	aconitase	3	1.82	56.98	0.0064*	NO	0.6667
**3.4. Pentose-phophate Pathway**							
gi|295666688	transaldolase	2	0.53		**	NO	NO
gi|295663567	6-phosphogluconolactonase	2	0.25	5.30	0.2191*	NO	0.6288
**4. CELL CYCLE AND DNA PROCESSING**							
gi|295658863	Cofilin/tropomyosin-type actin-binding family protein	2	18.754	1911.74	0.0076*	NO	NO
**5. PROTEIN FATE (folding, modification, destination)**							
gi|295672500	aminopeptidase	1	0.33		**	0.996	0.9401
gi|295657201	glutamate carboxypeptidase	2	0.79	51.03	0.0199*	NO	0.7370
gi|295658437	mitochondrial-processing peptidase subunit alpha	1	0.09		**	NO	0.6031
gi|295668481	peptidyl-prolyl cis-trans isomerase D	1	0.28	10.46	0.0325*	NO	0.8640
**6. PROTEIN SYNTHESIS**							
gi|295674311	eukaryotic translation initiation factor 5A	1	0.38	5.15	0.0092*	NO	NO
gi|295675019	elongation factor 2	1	2.54		**	NO	NO
gi|295668925	elongation factor 1-gamma 1	1	0.19		**	NO	0.9067
**7. UNCLASSIFIED PROTEINS**							
gi|295658312	L-PSP endoribonuclease family protein	1	2.02		**	NO	NO

1 NCBI database general information number (http://www.ncbi.nlm.nih.gov/).

2 Number of identified isoforms of protein in *Paracoccidioides, Pb01* mycelia phase secretome.

3 The average of amount of values of abundances of all identified isoforms used to statistical test.

4 The ratio of M to Y abundance in *Paracoccidioides* secretome.

5 ANOVA – statistically significant differences are considered with *p*<0.05 (*); proteins were not detected in yeast secretome (**).

6 Secretion prediction according to Signal P 3.0 server, the number corresponds to signal peptide probability (http://www.cbs.dtu.dk/services/SignalP/).

7 Secretion prediction according to Secretome P 2.0 server, the number corresponds to neural network that exceeded a value of 0.5 (NN-score **≥**0.50) (http://www.cbs.dtu.dk/services/SecretomeP/).

### 3.5. Extracellular proteins upregulated in *Paracoccidioides Pb*01 yeast cells

When comparing the secretome of mycelia and yeast cells, 24 proteins were identified that are preferentially secreted by yeast cells into the extracellular environment as depicted in Table 3 and Figure 3B. The differentially expressed proteins displayed fold changes ranging from 2.72 to 70672.37 when compared with mycelia. Most of these proteins play a role in cell rescue, cell defense, cell virulence, cell cycle, DNA processing, and energy production (Table 3). The proteins that play a role in cell rescue, defense and virulence, including heat shock protein 60 (fold change of 6.20), Hsp90 binding co-chaperone (fold change of 20.97), disulfide isomerase Pdi1 (fold change of 5.76), superoxide dismutase (fold change of 2.72), thioredoxin-like protein (fold change of 8.40) and hsp70-like protein (fold change of 7.91), were more abundant in the yeast secretome. The 2-methylcitrate synthase protein was significantly expressed in the yeast phase secretome with a fold change of 70672. Interestingly, several proteins were detected exclusively in the yeast phase, including glutathione S-transferase, energy production-associated enzymes such as pyruvate kinase and phosphoglycerate mutase, proteins that play a role in cell cycle and DNA processing, and proteins involved in cellular transport such as the vesicular-fusion protein, SEC17. The proteins expressed exclusively in the yeast secretome (compared with the mycelia) are summarized in Table S2.

**Table 3:** Preferentially secreted proteins by *Paracoccidioides Pb01* yeast cells.

General Information number (NCBI) 1	Protein description	Number of isoforms in yeast 2	Amount of isoform abundances 3	ratio of Y/M abundance 4	ANOVA (*p*-value) 5	SignalP Score ≥ 0.5 6	SecretomeP Score ≥ 0.5 7
**1. CELL RESCUE, DEFENSE and VIRULENCE**							
gi|295658865	heat shock protein 60	3	0.38	6.20	0.0121*	NO	NO
gi|295665077	Hsp90 binding co-chaperone (Sba1)	2	1.33	20.97	0.0013*	NO	NO
gi|295673162	disulfide isomerase Pdi1	1	0.20	5.76	0.0323*	0.988	0.899
gi|295667577	glutathione S-transferase Gst3	1	2.44		**	NO	0.5119
gi|295666684	Cu - Zn superoxide dismutase	2	0.46	2.72	0.0013*	NO	0.9184
gi|295659831	thioredoxin-like protein	2	2.98	8.40	0.0394*	NO	0.8876
gi|295659116	hsp70-like protein	6	596.89	7.91	0.0263*	NO	0.5000
**2. METABOLISM**							
**2.1. Amino Acid Metabolism**							
gi|225680243	Cobalamin-independent methionine synthase	1	0.04		**	NO	NO
**2.2. Secundary Metabolism**							
gi|295663891	2,5-diketo-D-gluconic acid reductase A	1	0.09		**	NO	NO
**3. ENERGY**							
**3.1. Glycolysis and Gluconeogenesis**							
gi|295662174	pyruvate kinase	1	0.09		**	NO	NO
**3.2. Oxidation of fatty acids**							
gi|295666179	2-methylcitrate synthase	8	645.67	70672.37	0.0039*	NO	0.5337
gi|295662074	3-hydroxybutyryl-CoA dehydrogenase	1	1.79		**	NO	NO
gi|295662032	enoyl-CoA hydratase	1	0.89		**	0.699	NO
**4. CELL CYCLE AND DNA PROCESSING**							
gi|295673184	actin-interacting protein	2	0.34	3.99	0.0018*	NO	0.9262
gi|295661300	DNA damage checkpoint protein rad24	2	0.85		**	NO	NO
gi|295667597	G4 quadruplex nucleic acid binding protein	1	1.96		**	NO	0.9401
gi|295668188	nuclear movement protein nudC	1	0.24		**	NO	0.7668
gi|295665468	nucleic acid-binding protein	2	18.32	19.49	0.0052*	0.982	0.5915
**5. PROTEIN FATE (folding, modification, destination)**							
gi|295660102	dipeptidyl-peptidase	3	0.27	3.47	0.0250*	NO	NO
gi|295660961	gamma-glutamyltranspeptidase	1	1.26		**	NO	NO
gi|295672926	proteasome component PRE4	1	0.77	15.82	0.0220*	NO	NO
**6. CELLULAR TRANSPORT, TRANSPORT FACILITIES AND TRANSPORT ROUTES**							
gi|295662829	vesicular-fusion protein SEC17	1	1.38		**	NO	NO
gi|295660305	cytochrome-c oxidase chain VI	2	1.49		**	0.9240	NO
gi|295670838	nucleolar transport factor 2	1	0.85		**	NO	0.9101

1 NCBI database general information number (http://www.ncbi.nlm.nih.gov/).

2 Number of identified isoforms of protein in *Paracoccidioides, Pb01* yeast phase secretome.

3 The average of amount of values of abundances of all identified isoforms used to statistical test.

4 The ratio of Y to M abundance in *Paracoccidioides* secretome.

5 ANOVA - statistically significant differences are considered with *p*<0.05 (*); proteins were not detected in mycelia secretome (**).

6 Secretion prediction according to Signal P 3.0 server, the number corresponds to signal peptide probability (http://www.cbs.dtu.dk/services/SignalP/).

7 Secretion prediction according to Secretome P 2.0 server, the number corresponds to neural network that exceeded a value of 0.5 (NN-score **≥**0.50) (http://www.cbs.dtu.dk/services/SecretomeP/).

### 3.6. Enzymatic activity correlates with proteomic data

**Figure 4:**
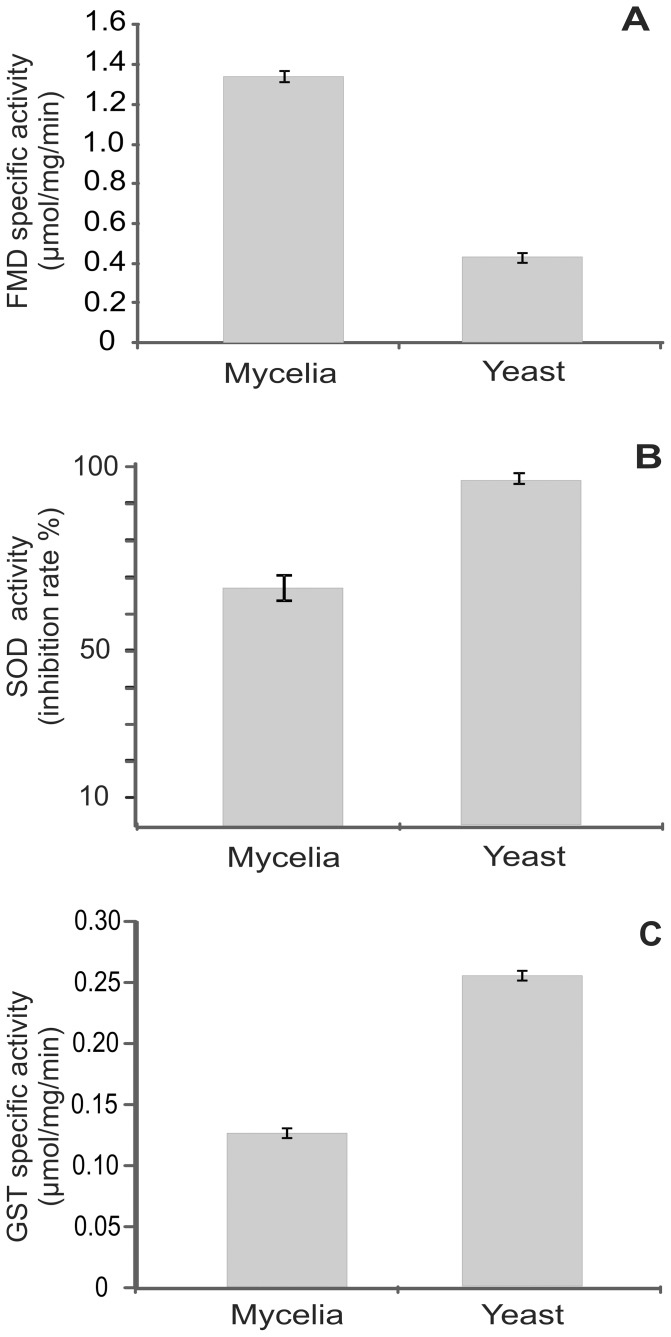
Enzymatic activity analysis validates the secretome data for *Paracoccidioides* mycelia and yeast cells. Activity assay results of (**A**) formamidase (FMD), (**B**) superoxide dismutase (SOD) and (**C**) glutathione S-transferase (GST) assessed for mycelia and yeast protein extracts. FMD activity was assessed by measuring the levels of ammonia released using a standard curve. The SOD and GST Assay Kit were used to determine SOD and GST enzymatic activity, respectively. The student's t test was used for statistical comparisons, and the observed differences were statistically significants (*p*≤0.05). The erros bars represent the standard deviation of three biological replicates.

To validate the significance of the proteomic results, enzymatic assays were performed with extracellular extracts for formamidase (FMD), superoxide dismutase (SOD) and glutathione S-transferase (GST). The differences observed with the 2D-gel analysis correlate with the enzymatic assays (Figure 4). The protein levels and enzymatic activity of FMD was increased in the mycelia secretome compared with the yeast phase as depicted in Table 2 and Figure 4, panel A. SOD and GST presented higher levels of activity in the yeast parasitic phase (Table 3 and Figure 4, panels B and C, respectively). For all the analyzed enzymes, the activity levels correlated with the proteomics data (Tables 2 and 3).

### 3.7. Protein isoforms and post-translational modifications

The 160 proteins/isoforms identified in the secretome of *Paracoccidioides Pb*01 are encoded by 86 different genes. Protein isoforms were identified for 37 of these gene products as depicted in Table S1. This observation suggests that many secreted proteins undergo post-translational modification. Table S3 presents the predicted post-translational modifications for the proteins identified in the mycelia and yeast secretomes. Hsp-70 like protein, heat shock SSC1, and 2-methylcitrate synthase represent the molecules displaying the highest number of identified isoforms (seven, eight and nine, respectively). The presence of the same protein displaying differential relative migration with 2D analysis may be associated with post-translational modification (PTM). To predict the putative PTMs, which explain the high isoform frequency, the most common S-T phosphorylation and lysine acetylation was included as a variable modification in the MASCOT search. The predicted PTMs were confirmed via manual spectral analysis as previously described [33]. In spite of the limitations associated with the MASCOT search for the PTM study, this strategy has been used previously to perform such analysis in organisms [42]. We observed an increase in peptide matches and sequence coverage when using those search criteria. Table S3 presents the predicted post-translational modifications for proteins in the mycelia and yeast secretomes. A total of 116 isoforms were analyzed using such criteria, and peptides displaying phosphorylation, acetylation or both PTMs were detected for 44 isoforms (Table S3). Serine/threonine phosphorylation was observed for 31 isoforms, while acetylation was observed for 30 isoforms (Table S4). The spectral analysis of isoform 46 of the heat shock protein, SSC1, revealed twelve phosphorylation and six acetylation sites. Many isoforms identified in the secretome displayed similar molecular weights and different *pI*s shifts. In contrast, a series of isoforms differed in both molecular weight and *pI*. This observation may be due to other PTMs, such as glycosylation, which were not analyzed because of the high complexity spectrum of the structures that glycan branches assume [43]. Further studies using analytical tools more appropriate for PTM analysis are required to confirm these putative findings.

### 3.8. Conventional and Non-conventional protein secretion by *Paracoccidiodes*

The *in silico* analysis indicated that 65% of the *Paracoccidioides* secretome included proteins/isoforms with known classical or non-classical secretion signals and that are actively exported to the extracellular space. Using the SignalP program, we found that 12.5% of the extracellular proteins/isoforms displayed putative signal peptide sequences (Table S1). In contrast, 52.5% of the identified extracellular proteins/isoforms were predicted, using the SecretomeP program, to be secreted by mycelia and yeast via a non-classical mechanism. Proteins/isoforms that were not predicted to be secreted by yeast and mycelia represented 56 of the 160 identified spots (35%) (Table S1). These data are in agreement with those reported by the Fungal Secretome Database (FSD), which describes that 58% of the 9,136 proteins expressed by the *Paracoccidioides Pb*01 genome are predicted to be Class NS (non-classically secreted), while 14% display a classical secretion signal (Class SP) and 28% of the proteins have not been classified (Figure S2).

### 3.9. Blocking canonical protein secretion in *Paracoccidioides Pb*01 yeast cells influences fungal adhesion/internalization by macrophages

**Figure 5:**
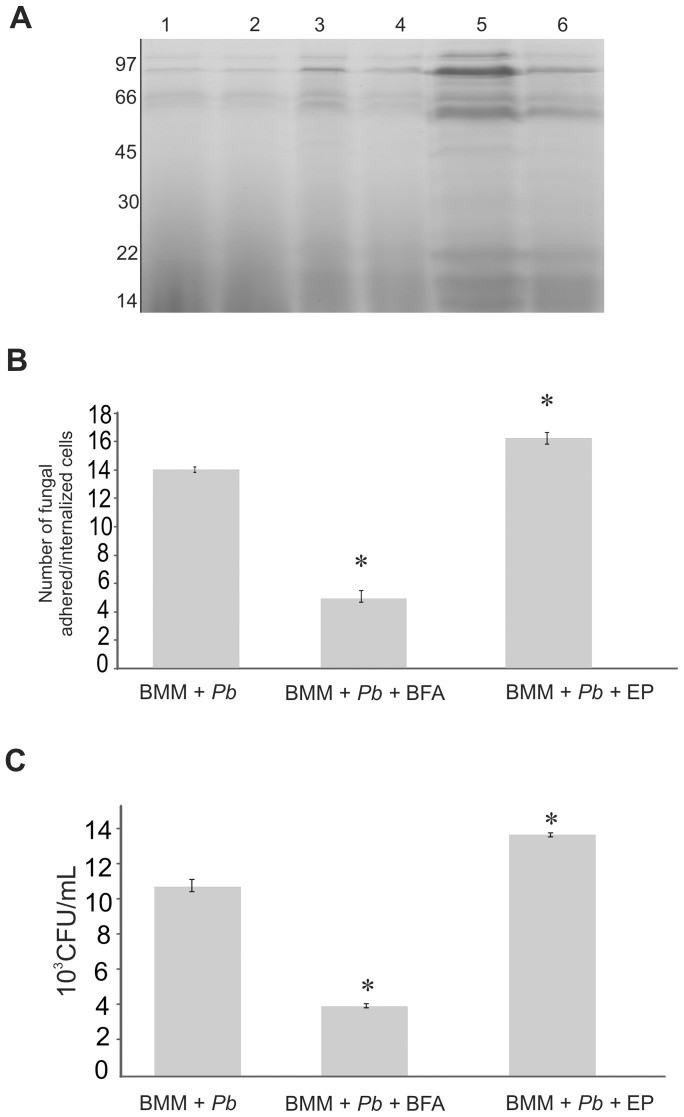
Blocking the conventional protein secretion pathway leads to a decrease in *Paracoccidioides* yeast cell phagocytosis. **A**- The protein profile of the cell-free supernatant samples reveals the effect of blocking the protein secretion pathway on yeast cells. *Paracoccidioides* yeast cells were cultivated in Fava Netto's liquid medium in either the absence (control) (lanes 1, 3 and 5) or presence of Brefeldin A (BFA) at 6 μg/mL (lanes 2, 4 and 6) for 6, 12 and 24 hours, respectively. The cell-free supernatant samples were prepared (as described in the Materials and Methods section), reduced to equal final volumes (1 mL), and processed for one-dimensional electrophoresis (SDS-PAGE). Thirty microliters of each sample was separated via SDS-PAGE and visualized using Coomassie brilliant blue staining. The numbers on the left side correspond to the molecular mass standard. **B**- The average number of internalized/adhered *Paracoccidioides* cells by macrophages was determined. Macrophages were infected with *Paracoccidioides* yeast cells, which were pre-cultivated previously without BFA (control), in the presence of BFA or the presence of concentrated culture supernatant containing extracellular proteins (EP). The adhered/internalized cells were analyzed as described in the materials and methods section. **C**- The number of viable yeast cells after phagocytosis by macrophages was evaluated by counting the number of colony forming units (CFUs). The results are representative of triplicate biological samples. Statistical significance (* *p*≤0.05) was determined by comparing the results with the control group.

Experiments were performed to investigate the effect of protein secretion on the adhesion/internalization of *Paracoccidioides* yeast cells by macrophages. As demonstrated in Figure 1A, yeast cell viability was over 90% during 24-hour incubation in the presence of the protein secretion inhibitor, Brefeldin A (BFA). Additionally, the growth rate of yeast cells in the presence of BFA was not altered during the incubation as depicted in Figure 1B. The yeast cell protein profile upon incubation with BFA was analyzed via SDS-PAGE using equal volumes of each extract (30 µL). As depicted in Figure 5A, BFA reduced the levels of proteins secreted by yeast cells starting from 12 hours with a stronger effect after 24 hours of incubation. Therefore, treatment with BFA at 24 hours was used for the adhesion/internalization experiments involving *Paracoccidioides Pb*01 and macrophages.

The number of internalized/adhered *Paracoccidioides* cells by macrophages was significantly decreased after yeast treatment with BFA for 24 hours compared with the control as shown in Figure 5, panel B. Additionally, the yeast cell treatment with BFA resulted in a decreased recovery of viable yeast cells in macrophages, (Figure 5, panel C). This finding reflects the consequence of the decrease in the number of internalized/adhered *Paracoccidioides* cells by macrophages with the addition of BFA, thereby suggesting that secretion inhibition decreases the association of macrophages with yeast cells. In contrast, the addition of concentrated culture supernatant containing *Paracoccidioides* extracellular proteins resulted in an increase in the number of internalized/adhered fungal cells by macrophages, as well as in increase in the recovery of viable yeast cells into macrophages (Figure 5, panels B and C, respectively).

The Brefeldin's effect on macrophages, which was evaluated by adding BFA to the co-cultivation, failed to cause a significant difference in the average number of internalization/adherence yeast cells by macrophages (data not shown). Figure S3 depicts microscopy analysis of *Paracoccidioides* adhesion and internalization by macrophages, which was used to determine the average number of internalized/adhered fungi cells in the control (panels A and B), in the presence of BFA (panels C and D) and in the presence of concentrated culture supernatant (panels E and F). The BFA treatment reduced the number of yeast cells adhered/internalized by macrophages, while the addition of extracellular proteins increased the rate of phagocytosis.

### 3.10. *Paracoccidioides Pb*01 *yeast* cell secreted proteins identified in macrophages

A nanoLC-MS/MS-based proteomics approach was employed to identify *Paracoccidioides Pb*01 yeast cell secreted proteins in *in vitro* cultured macrophages. A total of 18 *Paracoccidioides* proteins were identified in the cytoplasm of infected macrophages (Table 4), including enolase, heat shock proteins, translation elongation factors, DNA damage checkpoint proteins, and peptidyl-propyl cis-trans isomerase D. No *Paracoccidioides* proteins were identified in the negative control.

**Table 4:** *Paracoccidiodes, Pb*01 yeast cells secreted proteins in macrophages by nanoLC-MS/MS.

General Information Number (NCBI)^1^	Protein description	Protein Score	Matching peptides	Coverage (%)
gi|295666197	2 methylcitrate dehydratase	1.515.417	6	20.88
gi|226291035	ATP synthase subunit alpha	2.842.065	10	27.34
gi|295658821	ATP synthase subunit beta	5.217.957	20	54.39
gi|295661300	DNA damage checkpoint protein rad24	1.295.329	6	40.57
gi|295672736	DNA damage checkpoint protein rad24	1.222.672	5	36.16
gi|295671178	elongation factor 1 alpha	8.897.567	13	33.26
gi|295668925	elongation factor 1 gamma 1	2.384.459	9	25.06
gi|146762537	enolase	14668.68	12	45.43
gi|295658119	glyceraldehyde 3 phosphate dehydrogenas	3201.45	8	41.95
gi|226278527	10 kDa heat shock protein	20459.57	5	45.63
gi|60656557	heat shock protein	1483.16	18	20.57
gi|295658865	heat shock protein 60	8.995.715	23	47.56
gi|295671569	heat shock protein SSC1	2.446.586	13	22.5
gi|295659116	Hsp70-like protein	8.972.324	22	47.09
gi|295658218	malate dehydrogenase	1.135.156	10	28.79
gi|295673937	malate dehydrogenase	4.400.604	13	65.88
gi|295666938	nucleoside diphosphate kinase	14429.06	5	38.82
gi|295668481	peptidyl-prolyl cis-trans isomerase D	1.868.925	8	27.08

1 NCBI database general information number (http://www.ncbi.nlm.nih.gov/).

### 3.11. Immunoblot analysis of macrophage lysates

We aimed to determine whether *Paracoccidioides* expresses constituents of the extracellular proteome during infection in cultured macrophages by examining the secretion of candidate proteins via immunoblot analysis. The immunoblot analysis of macrophage lysates revealed that *Paracoccidioides* yeast cells secrete enolase (*Pb*Eno) and triosephosphate isomerase (*Pb*Tpi) in infected macrophages (Figure Suplementary 4, line 2, panels A and B, respectively). The pre-incubation of *Paracoccidioides* yeast cells with Brefeldin A, a protein secretion inhibitor, displayed a lack of *Pb*Eno and *Pb*Tpi expression in macrophages (Figure Suplementary 4, line 1, panels A and B, respectively).This finding is most likely due to the reduced number of internalized/adhered *Paracoccidioides* cells by macrophages during BFA treatment as showed in Figure S3. The negative control represents the non-infected macrophage lysate which indicated that those antibodies did not target mouse proteins (Figure Suplementary 4, line 3).

### 3.12. Comparative analysis between *Paracoccidioides Pb*01 extracellular proteins with orthologue proteins found in pathogenic fungi secretomes

We compared the ORFs sequences of the extracellular proteins identified in *Paracoccidioides Pb*01 yeast cells with the orthologues previously reported in the secretome of *Paracoccidioides Pb18* [27], *H. capsulatum* [10], [18], *C. neoformans* [19] and *A. fumigatus* [37] as demonstrated in Table S5 and Figure S5. *P. brasiliensis* (*Pb*18) and *P. lutzii* (*Pb*01) displayed specific variations in the yeast phase secretome. A total of 63 proteins/isoforms detected in the secretome of yeast cells in *Pb*01 (57%) have also been described for the secretome of *Pb*18. Among the common proteins, several enzymes were identified including those involved in the metabolism of carbohydrates and lipids and heat shock proteins. Additionally, 47 proteins/isoforms, from a total of 110, were not common to *Pb*01 and *Pb*18, thereby suggesting differences in gene expression/secretion between the two species of *Paracocidioides.* Proteins exclusively identified in the secretome of *Pb*01 yeast cells (compared with *Pb*18) included glutathione reductase and glutathione S transferase. Eighty eight identified extracellular proteins in *Paracoccidioides Pb*01 (80%) had orthologues that have been previously described in fungal secretomes (Table S5 and Figure S5), while 22 extracellular proteins were exclusively found in the *Pb*01 yeast cells. We found that 59% of the extracellular proteins identified in *Pb*01 (65 proteins/isoforms) had orthologues in *H. capsulatum* (Suplementary Figure 5). Among these proteins, 45 proteins/isoforms were also found in *Pb*18 (Table S5). Concerning to *C. neoformans* and *A. fumigatus* the small number of proteins described in the secretomes may account to the reduced number in orthologues to *Pb*01.

## Discussion

In the current study, the secretome profile of *Paracoccidioides Pb*01 was described for the first time. Using a proteomic analysis, 160 proteins/isoforms were identified. Additionally, the proteomic analysis revealed that the secretome consisted of 20 extracellular proteins/isoforms of the classical secretory pathway and 84 extracellular proteins/isoforms secreted via non-classical secretory pathways. Secretion involves a vesicular transport mechanism, thereby suggesting that an exosome-like structure represents a conserved mechanism for the release of molecules by fungal cells [17]–[19]. Moreover, recent results have demonstrated vesicle-dependent secretion in *Paracoccidioides* [27].

A total of 28 proteins, which correspond to 47 isoforms (29.37% of the total identified proteins/isoforms) were described as constitutively (no difference in protein levels) secreted by *Paracoccidioides Pb*01 yeast and mycelia. This group includes proteins that are involved in cellular processes such stress response, metabolism, energy and protein fate. Two heat shock proteins/isoforms were constitutively secreted by mycelia and yeast cells. Heat shock proteins have also been reported to be involved in the increase of the export of proteins via non-classical mechanisms in eukaryotic cells [44]–[46]. Therefore, we hypothesize that heat shock proteins may be involved in protein exportation by *Paracoccidioides*.

We analyzed the *Paracoccidioides Pb*01 yeast cells and mycelia secretomes and performed a comparative analysis of the changes in protein profiles between the two phases. In the present study, without any enrichment strategies, 118 and 110 protein/isoforms (Table S1) were detected in *Paracoccidioides* mycelia and yeast, respectively. The proteomic analysis resulted in the identification of 160 non-redundant proteins/isoforms, of which 98 are differentially expressed in the two phases.

Formamidase was found to be preferentially secreted by mycelia. This result was consistent with the enzymatic activity assay results, as formamidase activity was significantly higher in mycelia compared with yeast cells. Formamidase was also detected in *H. capsulatum* extracellular vesicles [18]. Proteins involved in amino acid metabolism, such as serine hydroxymethyl transferase, were preferentially secreted by mycelia. In microorganisms [47] and plants [48], this enzyme plays an important role in conferring tolerance to salinity stress by generating amino acids, such as serine, and ammonium quaternary compounds, such as glycine betain. These molecules protect secreted proteins and other molecules against salinity stress by acting as non-enzymatic chaperones [48]. In *Paracoccidioides*, this enzyme may play role in the protection against environmental stress during the mycelia phase.

Additionally, proteins that play a role in cell defense, such as peroxisomal catalase, glutathione reductase, thioredoxin reductase and translationally-controlled tumor protein (TCTP), were identified in the *Paracoccidioides* mycelia secretome. The increased secretion of enzymes involved in cell defense may reflect a need for increased protection against stress. The mycelia phase of *Paracoccidioides* occurs in the soil and can be exposed to many environmental stresses, such as high temperature and solar radiation and reactive oxygen species that can emerge from external sources [49]. Additionally, reports have implicated the involvement of TCTP in the prevention of cell death by modulating the anti-apoptotic activity of Mcl-1 [50]. The results suggest the secretion of proteins/enzymes required to protect the saprobe phase from environmental insults.

Glutathione S-transferase (GST) was found to be preferentially secreted by *Paracoccidioides Pb*01 yeast compared with the mycelia secretome. GST represents a group of detoxification enzymes, which are involved with protection against oxidative stress and the detoxification of xenobiotics and heavy metals [51], [52]. In agreement with the proteomic data, the GST activity was significantly higher in the yeast cell secretome compared with that of the mycelia. Additionally, superoxide dismutase was preferentially secreted by *Paracoccidioides Pb*01 yeast cells. The *H. capsulatum* extracellular superoxide dismutase (SOD3) has been shown to be preferentially expressed by yeast cells compared with the expression by non-pathogenic mycelia [10]. SOD3 specifically protects against extracellular reactive oxygen species and facilitates *H. capsulatum* pathogenesis by detoxifying host-derived reactive oxygen species [53]. Proteomic analysis results displayed eight isoforms of 2-methylcitrate synthase in *Paracoccidioides* yeast, which were predicted to be exported via a non-classical secretory pathway. The statistical analyses revealed 2-methylcitrate synthase was secreted at significant levels (fold change of 70672.37). This protein was also previously demonstrated in the extracellular vesicles of *H. capsulatum* and *Paracoccidioides Pb*18 [18], [27], although its function at the extracellular environment had not been established. DNA damage checkpoint protein (14-3-3 protein family) was also preferentially expressed by the *Paracoccidioides Pb*01 yeast secretome. The 14-3-3 protein regulates a diverse range of cell signaling pathways by forming protein-protein interactions and modulating the protein function in eukaryotic cells [54]. Moreover, 14-3-3 protein modulates vesicular trafficking and exocytosis, as displayed in different experimental systems [55], [56]. This protein was also detected in the extracellular environment by the proteomic analysis of various fungi, such as *C. neoformans* [19], *H. capsulatum* [10], [18] and *Paracoccidioides, Pb*18 [27]. Proteins that function in cell rescue, defense and virulence were more abundant in the yeast secretome, which included proteins involved in stress response and detoxification, such as heat shock proteins, disulfide isomerase Pdi1, glutathione S-transferase Gst3 and superoxide dismutase. During the infection process by the pathogenic yeast, the release of reactive oxygen species (ROS) by the immune effector cells plays an important role in killing microbes [57]. Therefore, the response to stress is likely to be an important virulence attribute of this pathogenic fungus.

Proteomics studies can be heavily influenced by the growth conditions. As example, *Botrytis cinerea* was shown to secrete a wide array of enzymes and there are significant changes in the relative abundance and composition of secreted enzymes in a substrate dependent manner [58]. We have used a carbohydrate-and peptide rich medium in our studies that could explain the abundance of some glycolysis and TCA-cycle proteins in the secretomes of yeast and mycelia. Although this consideration, the yeast and mycelia phase grown in the same medium secrete differentially a wide array of proteins/enzymes, suggesting that those molecules are likely tailored to benefit the saprobe and pathogenic yeast phase.

Despite its limitations, 2D gel electrophoresis remains a powerful method for global inspection of post-translational modification, as it is associated with shifts in protein molecular mass and charge. Among the secreted proteins identified in *Paracoccidioides Pb*01 mycelia and yeast cells, 37 appeared in more than one spot on the 2D-gel, thereby totaling 116 identified isoforms (72.5% of the total). We speculated that some of the *pI* or molecular mass changes were caused by PTM. Spectral analysis combined with a PTM MASCOT search revealed that 39% of the detected isoforms harbor serine/tyrosine phosphorylation and/or lysine acetylation sites. Eight of the nine isoforms of 2-methylcitate synthase were found to be phosphorylated, which correlates with the decrease in *pI*. We also identified 30 acetylated proteins out of 116 isoforms, and all isoforms of the hsp70-like proteins harbored at least five acetylated peptides. The *pI* shifts in those molecules correlates with those observed in proteins derived from breast cancer cell lines, in which the *pI* shifts were consistent with the number and type of modifications [59]. The PTM may be associated with novel functions of intracellular proteins located outside of the fungal cells. However, the impact of these modifications on pathogenesis, environmental adaptation, nutrient uptake and morphological maintenance/changes has not yet been clarified and would require a more comprehensive global PTM analysis and more detailed investigation of the impact of each modification on the function of each protein modification [42].

We investigated the function of conventional protein secretion in the *Paracoccidioides* macrophage interaction. The addition of BFA to the culture medium led to a decrease in secreted proteins, significantly inhibited the macrophage/fungus interaction (assessed by the adherence and internalization of yeast cells) and caused a reduction in viable cell recovery from macrophages. Consequently, it may be hypothesized that the inhibition of macrophage functions might facilitate the survival of the pathogen within the phagocytic cell [39], [60]. The *Paracoccidioides* yeast cells is known to both be phagocytosed by and multiply inside macrophages [61]. The involvement secreted proteins in the uptake of *Paracoccidioides* by macrophages has been described [61]. The gp43, the main antigenic molecule secreted by *Paracoccidioides*, participates during the initial steps of attachment of the fungus to macrophages was demonstrated when polyclonal antibodies directed to some gp43 epitopes induced a marked decrease in the phagocytic indexes of macrophages challenged with *Paracoccidioides* yeast cells [61]. These data attest the function of gp43 extracellular proteins in the adherence of the fungus and uptake by macrophages. Similarly, we observed that the addition of BFA significantly inhibited the yeast cells internalization by macrophages. In contrast, the addition of concentrated culture supernatant significantly increased the interaction of macrophages with yeast cells, thereby suggesting that the secreted proteins released by *Paracoccidioides* yeast cells exert some biological activity. A similar ability has been described for *Leishmania-*secreted vesicles during infection, which deliver effectors that mediate parasite invasion favoring survival in the host [62], [63]. Furthermore, *Leishmania* exosomes and exosomal proteins have been detected in the cytoplasmic compartment of infected macrophages [64].

Specialized secretion systems are used by human pathogens to export virulence factors into host target cells [64]–[66]. In this report, we present evidence that *Paracoccidioides* also releases effector proteins in macrophages; however, this mechanism remains elusive. We identified 18 secreted proteins in infected macrophages, including the protein orthologues heat shock protein 10, heat shock protein 70, DNA damage checkpoint protein (14-3-3 protein) and elongation factor 1 alpha (EF-1α), which have been previously described as exosomal proteins released into infected macrophages by *Leishmania* [63], [64], thereby suggesting that these proteins may be effectors present in the host cytosol after the infection by macrophages has been established. Moreover, it has been described that the *Leishmania* protein, EF-1α, accesses the host cell cytosol and activates multiple host protein-tyrosine phosphatases (PTPs), which negatively regulate interferon-γ (INF-γ) signaling, thereby preventing effective expression of the macrophage microbicidal arsenal including TNF-α and nitric oxide (NO) production. Additionally, heat-shock results in an increase in exosomal vesicles release into the host cell by *Leishmania* [67]. Taken together, these results suggest that *Paracoccidioides* extracellular proteins may be important for fungal survival within macrophages. Therefore, we hypothesized that proteins secreted by *Paracoccidioides* may facilitate the survival of the fungus within the host, at least during the initial phase of infection, thereby reinforcing the concept that secreted proteins released by pathogenic fungi play a crucial role in pathogenesis and virulence [11], [68]. We hypothesized that the *Paracoccidioides* exoproteome may modulate both signaling pathways and function of the macrophages, thereby creating an environment permissive for early infection as has been described for other intracellular pathogens, such as *Leishmania* [63], [67], [69]; *H. capsulatum* [70], [71] *; C. neoformans* [72]; *Candida glabrata* [65] and *C. albicans* [73].

We found that *Paracoccidioides* secrete various types of proteins, including enzymes, heat shock proteins and many conventional cytosolic factors. Although we cannot completely rule out cellular lyses, the secretome samples were assessed to ensure that cell lyses did not influence the extracellular protein profiles. Some of the proteins here described have been detected in the extracellular environment in many fungi by different groups [9], [10], [19], [27], thereby supporting the suggestion that they are indeed exported from intact cells. Interestingly, cytosolic enzymes, such as enolase and glyceraldehyde-3-phosphate dehydrogenase (GAPDH), exert both enzymatic activity and alternative extracellular functions in *Paracoccidioides.* These enzymes have been found in the cytoplasm and at the cell wall of the *Paracoccicioides* yeast cells [22], [74]. The cell wall-associated GAPDH binds to extracellular matrix components and mediates the attachment and internalization of the *Paracoccidioides* yeast cells to host tissues, thereby potentially playing a role in the establishment of disease [74]. *Paracoccidioides* recruits plasminogen and activates the plasminogen fribrinolytic system in a process mediated by the cell wall-localized enolase, which potentially plays a role in the establishment of PCM [22]. Therefore, cytosolic enzymes may represent ‘moonlighting’ proteins functioning in the extracellular environment [13], [75].

Additionally, it is important to note that 63 of the 110 identified proteins/isoforms in the *Pb*01 yeast secretome displayed orthologues in the *Pb*18 secretome. The difference in the number of genes between *Pb*01 and *Pb*18 [5] may explain the variations in the protein profiles. This may also be because they were sub-cultured using different media (Fava-Netto for *Pb*01 and modified YPD medium for *Pb*18). Additionally, the application of varying proteomic methodologies to analyze the extracellular proteins of *Pb*01 and *Pb*18 may give rise to the variations found in the secretomes between the two members of the *Paracoccidioides* genus. The fact that members of the phylogenetic groups of *Paracoccidioides* produce varying combinations of extracellular yeast phase proteins is not unexpected; the pools of extracellular proteins secreted by different *Histoplasma* strains are also distinct from one another [76]. In conclusion, the results suggest that different strains may utilize different strategies for survival and pathogenesis.

## Conclusion

We have identified abundant proteins expressed in *Paracoccidioides* yeast and mycelia secretomes. Several extracellular proteins have also been described in the secretome of other organisms using different methodologies, which is important for the validation of our findings. Strikingly, many proteins do not use the classical secretory pathways, and many proteins most likely exert other activity once secreted. Moreover, the data clearly indicate that *Paracoccidioides Pb*01 predominantly uses non-classical targeting mechanisms to direct protein export. Our findings display the potential role that extracellular proteins play in fungus survival in the host and may lead to the description of molecules that function as virulence factors.

## Supporting Information

Figure S1 **Enzyme Class (EC) of identified proteins.** According to the Nomenclature Committee of the International Union of Biochemistry and Molecular Biology (NC-IUBMB), the identified enzyme-like proteins were grouped into six classes (Ligase, Isomerase, Lyase, Hydrolase, Transferase and Oxidoreductase).
(TIF)

Figure S2 **Prediction of protein secretion in** ***Paracoccidioides*** **.** All identified proteins were submited for *in silico* analysis using the SignalP 3.0 (http://www.cbs.dtu.dk/services/SignalP/) and SecretomeP 2.0 (http://www.cbs.dtu.dk/services/SecretomeP/) programs. The results obtained for the *Paracoccidioides* mycelia and yeast secretomes were grouped into classes and compared with the data provided in the Fungal Secretome Database (FSD) (http://fsd.riceblast.snu.ac.kr). Class **SP** includes proteins that were predicted by SignalP 3.0, Class **NS** represents proteins secreted via a non-conventional pathway, which were predicted by SecretomeP 2.0, and **NC** represents proteins that were not classified.
(TIF)

Figure S3 **Microscopy of adhered/internalized** ***Paracoccidioides*** **yeast cells by macrophages (magnification 1600X).** The yeast cells adhered and internalized (panel A) or internalized (panels A–F). The arrows indicate internalized yeast cells, and the asterisk indicates adhered yeast cells. The glass coverslips, which were cultivated in the absence of BFA (panels A and B), the presence of BFA (panels C and D) or the presence of concentrated culture supernatant (panels E and F) as described in the Materials and Methods section, were fixed with methanol, stained by Giemsa and visualized via electronic microscopy.
(TIF)

Figure S4 **Immunoblot analysis of** ***Paracoccidioides*** **-secreted proteins inside macrophages.** The tested samples included the lysate of infected macrophages with *Paracoccidioides* yeast cells treated previously with Brefeldin A (line 1), the lysate of infected-macrophages with *Paracoccidioides* yeast cells (line 2) and the lysate of non-infected macrophages as a negative control (line 3). **A-** Immunoblot analysis of secreted proteins in infected macrophages probed with enolase antibody (47 kDa). **B-** Immunoblot analysis of triosephosphate isomerase (29 kDa). **C-** Membranes stained with Ponceau red showing the protein profile of the tested samples (lines 1–3).
(TIF)

Figure S5 **Comparative analysis between** ***Paracoccidioides Pb*** **01 extracellular proteins and orthologous proteins found in pathogenic fungi secretomes.** The bar graph showing the proteins identified in the secretome of *Paracoccidioides Pb*01, *Paracoccidioides Pb*18 [27], *Histoplasma capsulatum* [10], [18], *Cryptococcus neoformans* [19] and *Asperillus fumigatus* [37] of various pathogenic fungi. The gray bars represent the number of extracellular orthologous proteins that overlap between *Paracoccidiodies Pb*01 yeast cells and the analyzed species. The white bars represent the extracellular proteins that not do overlap with *Paracoccidioides Pb*01 yeast cells.
(TIF)

Table S1 **Secreted proteins/isoforms by** ***Paracoccidioides Pb01*** **.** **^1^** Spots numbers indicated in Figure 2. **^2^** NCBI database general information number (http://www.ncbi.nlm.nih.gov/). **^3^** Molecular Mass in kDa (theoretical/experimental). **^4^** Isoelectric point (theoretical/experimental). **^5^** Mascot MS/MS score for fragmentation data (http://www.matrixscience.com). **^6^** Number of matched peptides (MS/MS). **^7^** Protein Expression in M: mycelia phase; Y: Yeast phase and C: protein common to the two fungal phases or protein with no differential expression. **^8^** ANOVA test - statistically significant differences are considered with *p*<0.05 (*); protein found at just one fungal phase (**). **^9^** Secretion prediction according to Signal P 3.0 server. The number corresponds to signal peptide probability (Score **≥**0.5) (http://www.cbs.dtu.dk/services/SignalP/). **^10^** Secretion prediction according to Secretome P 2.0 server, the number corresponds to neural network that exceeded a value of 0.5 (NN-score **≥**0.50) (http://www.cbs.dtu.dk/services/SecretomeP/). **^11^** Enzyme Classification recommended by Nomenclature Committee of the International Union of Biochemistry and Molecular Biology (NC-IUBMB). • adhesin-like proteins predicted by Faapred web server (http://bioinfo.icgeb.res.in/faap/query.html).
(DOC)

Table S2 **Exclusive proteins secreted by** ***Paracoccidioides Pb*** **01 yeast and mycelia.** **^1^** Spots numbers indicated in Figure 2. **^2^** NCBI database general information number (http://www.ncbi.nlm.nih.gov/). **^3^** Number of identified isoforms of protein in each *Paracoccidioides, Pb*01 phase secretome. **^4^** The average of amount of values of abundances of all identified isoforms. **^5^** Protein Expression in M: mycelia phase; Y: Yeast phase. **^6^** Secretion prediction according to Signal P 3.0 server, the number corresponds to signal peptide probability (http://www.cbs.dtu.dk/services/SignalP/). **^7^** Secretion prediction according to Secretome P 2.0 server, the number corresponds to neural network that exceeded a value of 0.5 (NN-score **≥**0.50) (http://www.cbs.dtu.dk/services/SecretomeP/).
(DOC)

Table S3 **Predicted post-translational modifications of identified protein isoforms in mycelia and yeast secretomes.** **^1^** Protein Expression in M: mycelia phase; Y: Yeast phase and C: protein common or protein with no differential expression. **^2^** PTM – Post translational modifications: acetyl (k) – lysine acetylation; phospo (S-T-Y) – serine, threonine and tyrosine phosphorylation. **^3^** Values returned by MASCOT search tool using none or specific variable modifications.
(DOC)

Table S4 **Spectral identification of post-translational modification in identified protein/isoforms.** **^1^** PTM – Post translational modifications: acetyl (k) – lysine acetylation; phospo (S-T-Y) – serine, threonine and tyrosine phosphorylation. **^2^** Peptide localization in protein sequence. **^3^** Theoretical peptide molecular mass without any PTM. **^4^** Obtained experimental mass by spectral analysis.
(DOC)

Table S5 **Comparative analysis of secreted proteins/isoforms by** ***Paracoccidioides Pb*** **01 yeast cells with others pathogenic fungal secretomes.** **^1^** Spots numbers indicated in Figure 2. **^2^** NCBI database general information number of *Paracoccidioides Pb*01 (http://www.ncbi.nlm.nih.gov/). **^3^** Accession number of orthologues present in the *Paracoccidioides Pb*18 secretome (Vallejo et al, 2012). **^4^** Accession number of orthologues present in the *Histoplasma capsulatum* secretome (Albuquerque et al, 2008; Holbrook et al, 2011). **^5^** Accession number of orthologues present in the *Cryptococcus neoformans extracelular vesicles* (Rodrigues et al, 2008). **^6^** Accession number of orthologues present in the *Aspergillus fumigatus* secretome (Wartenberg et al, 2011).
(DOC)
