# Supplementary figures and images for: Analysis of the Secretomes of Paracoccidioides Mycelia and Yeast Cells
Source: PLoS One. 2012 Dec 18;7(12):e52470. doi: 10.1371/journal.pone.0052470 (PMC3525554; doi:10.1371/journal.pone.0052470)

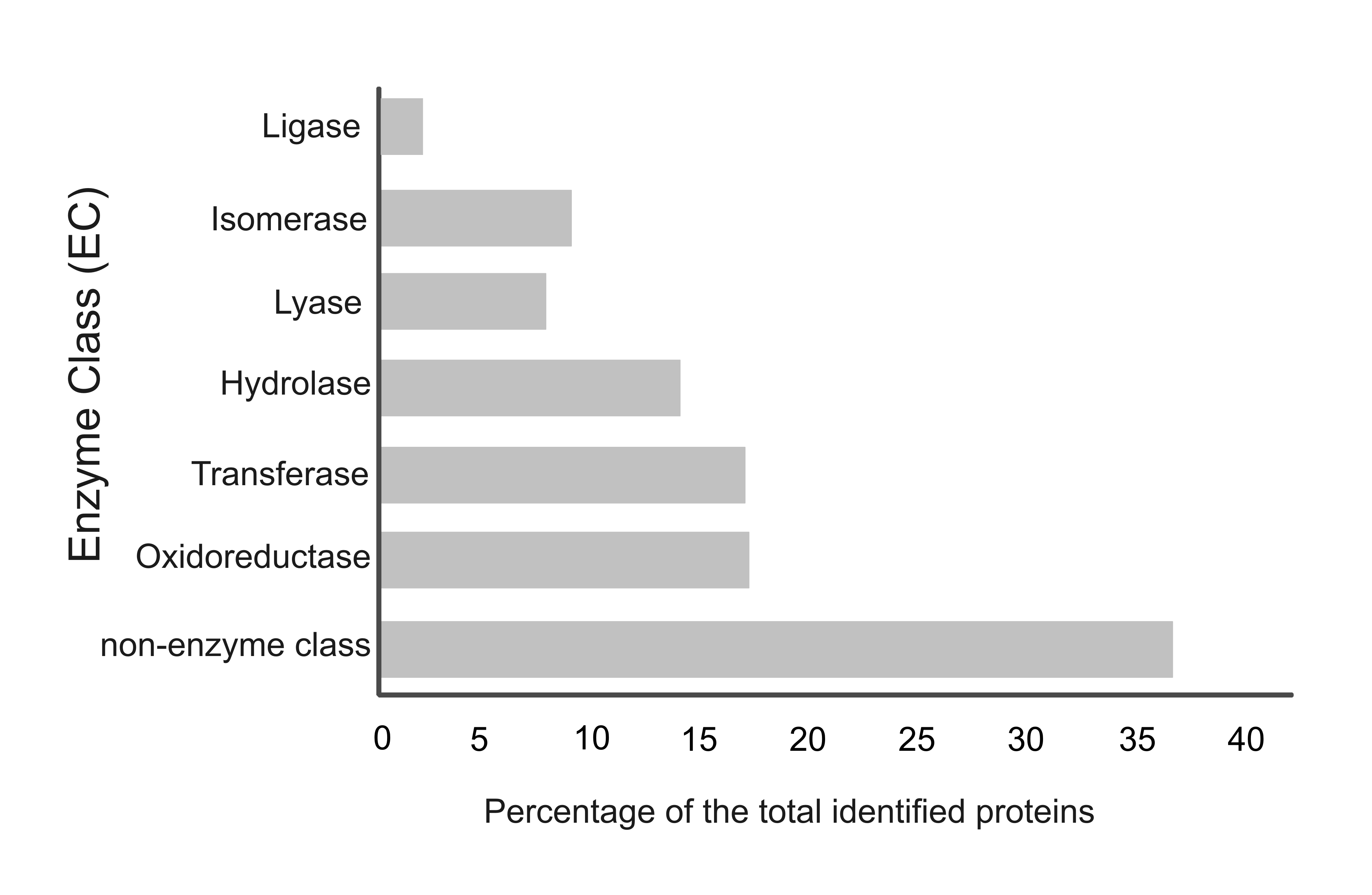

Supplement: Figure S1 — Enzyme Class (EC) of identified proteins. According to the Nomenclature Committee of the International Union of Biochemistry and Molecular Biology (NC-IUBMB), the identified enzyme-like proteins were grouped into six classes (Ligase, Isomerase, Lyase, Hydrolase, Transferase and Oxidoreductase). (TIF) [file pone.0052470.s001.tif]

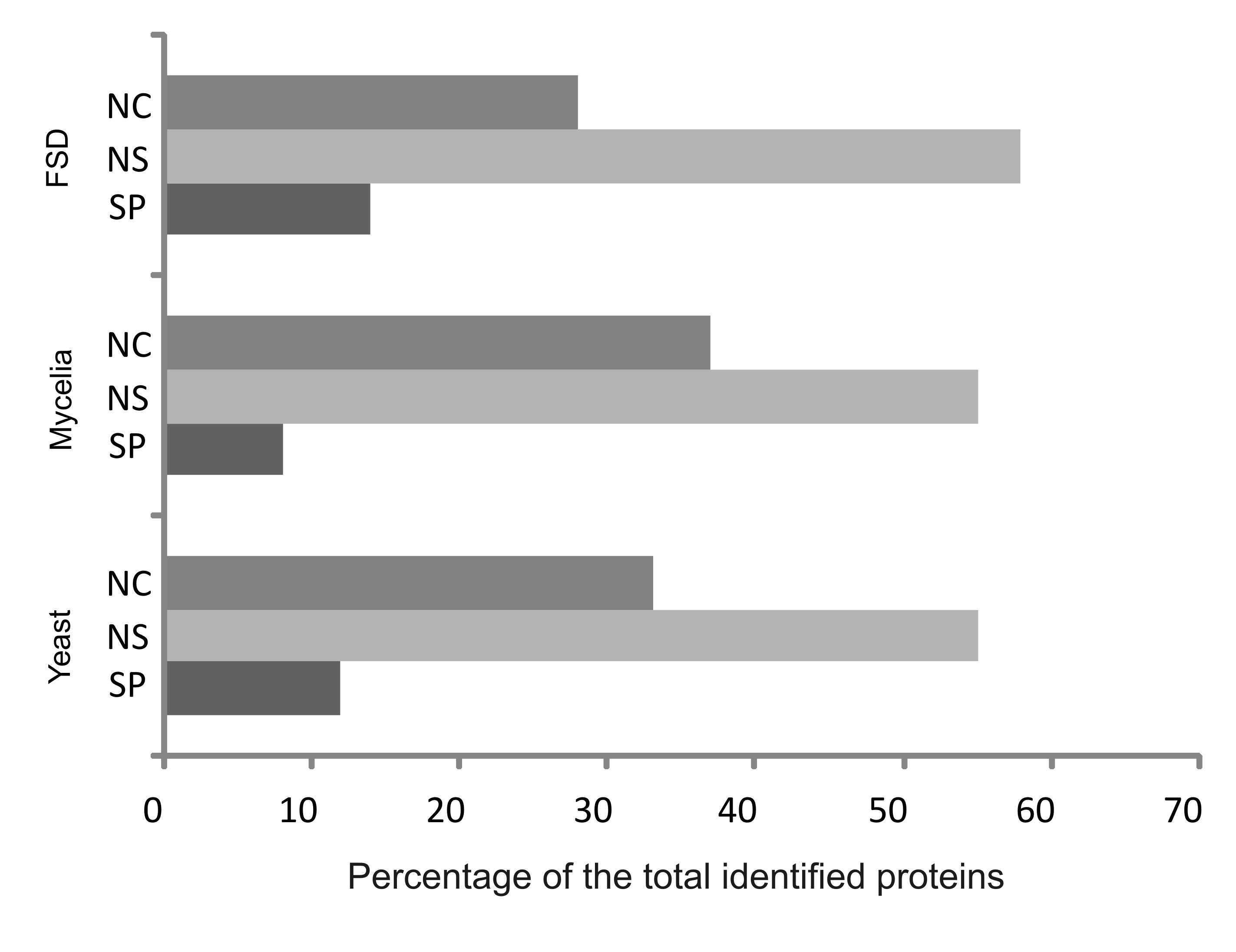

Supplement: Figure S2 — Prediction of protein secretion in Paracoccidioides . All identified proteins were submited for in silico analysis using the SignalP 3.0 (http://www.cbs.dtu.dk/services/SignalP/) and SecretomeP 2.0 (http://www.cbs.dtu.dk/services/SecretomeP/) programs. The results obtained for the Paracoccidioides mycelia and yeast secretomes were grouped into classes and compared with the data provided in the Fungal Secretome Database (FSD) (http://fsd.riceblast.snu.ac.kr). Class SP includes proteins that were predicted by SignalP 3.0, Class NS represents proteins secreted via a non-conventional pathway, which were predicted by SecretomeP 2.0, and NC represents proteins that were not classified. (TIF) [file pone.0052470.s002.tif]

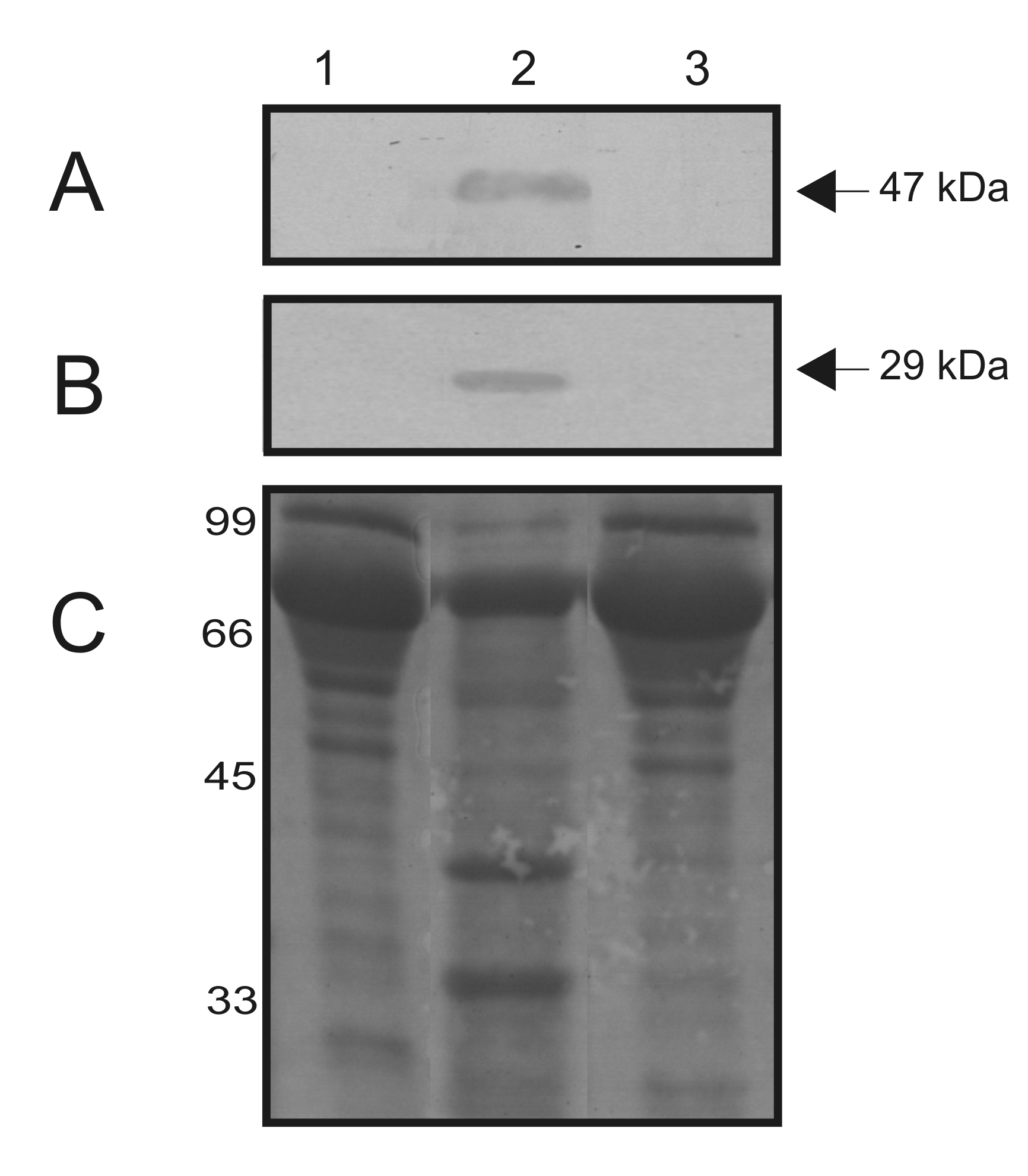

Supplement: Figure S4 — Immunoblot analysis of Paracoccidioides -secreted proteins inside macrophages. The tested samples included the lysate of infected macrophages with Paracoccidioides yeast cells treated previously with Brefeldin A (line 1), the lysate of infected-macrophages with Paracoccidioides yeast cells (line 2) and the lysate of non-infected macrophages as a negative control (line 3). A- Immunoblot analysis of secreted proteins in infected macrophages probed with enolase antibody (47 kDa). B- Immunoblot analysis of triosephosphate isomerase (29 kDa). C- Membranes stained with Ponceau red showing the protein profile of the tested samples (lines 1–3). (TIF) [file pone.0052470.s004.tif]

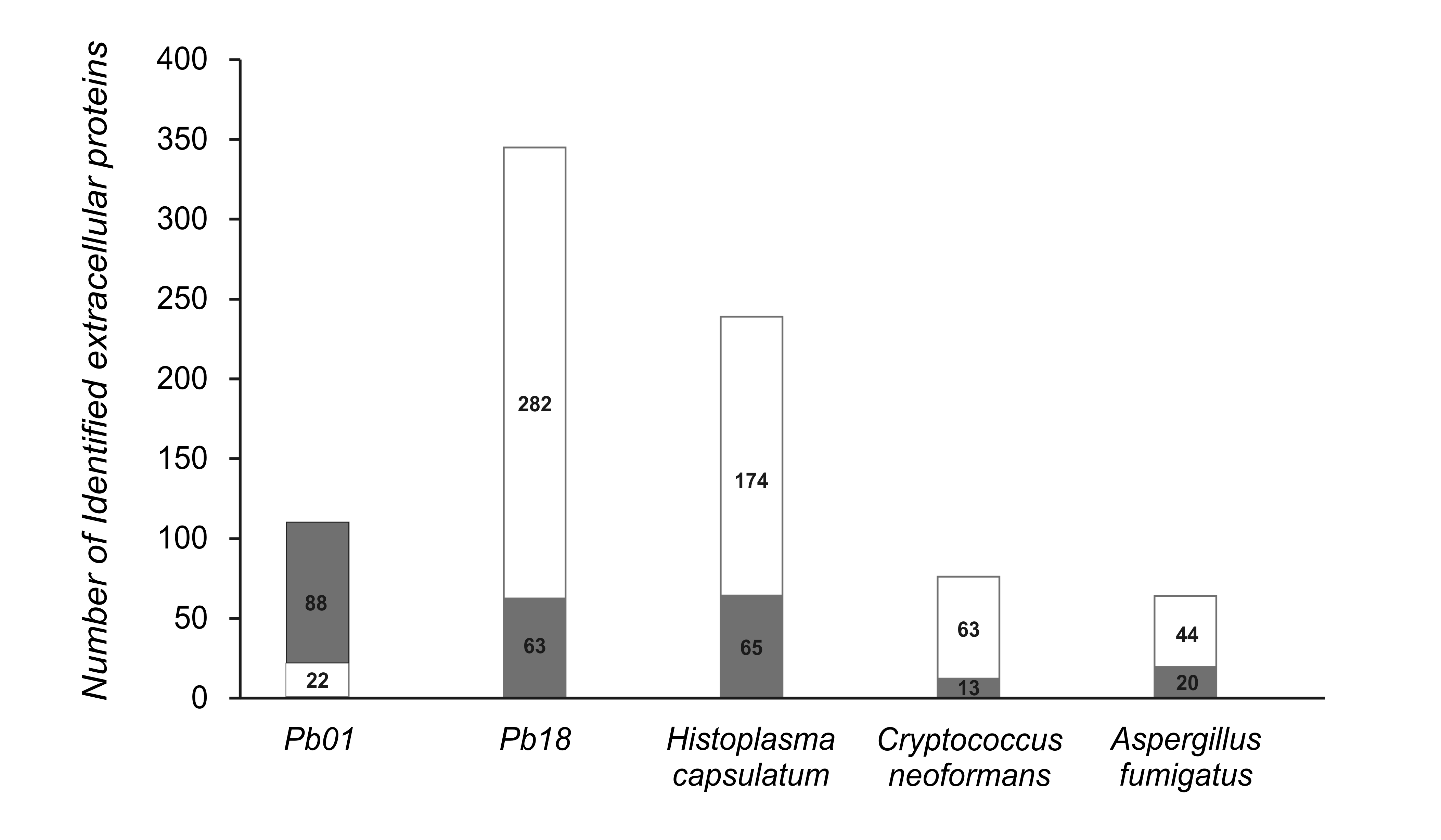

Supplement: Figure S5 — Comparative analysis between Paracoccidioides Pb 01 extracellular proteins and orthologous proteins found in pathogenic fungi secretomes. The bar graph showing the proteins identified in the secretome of Paracoccidioides Pb01, Paracoccidioides Pb18 [27], Histoplasma capsulatum [10], [18], Cryptococcus neoformans [19] and Asperillus fumigatus [37] of various pathogenic fungi. The gray bars represent the number of extracellular orthologous proteins that overlap between Paracoccidiodies Pb01 yeast cells and the analyzed species. The white bars represent the extracellular proteins that not do overlap with Paracoccidioides Pb01 yeast cells. (TIF) [file pone.0052470.s005.tif]
