# Supplementary material for: Analysis of the Secretomes of Paracoccidioides Mycelia and Yeast Cells
Source: PLoS One. 2012 Dec 18;7(12):e52470. doi: 10.1371/journal.pone.0052470 (PMC3525554; doi:10.1371/journal.pone.0052470)
Supplement: Table S1 — Secreted proteins/isoforms by Paracoccidioides Pb01 . 1 Spots numbers indicated in Figure 2. 2 NCBI database general information number (http://www.ncbi.nlm.nih.gov/). 3 Molecular Mass in kDa (theoretical/experimental). 4 Isoelectric point (theoretical/experimental). 5 Mascot MS/MS score for fragmentation data (http://www.matrixscience.com). 6 Number of matched peptides (MS/MS). 7 Protein Expression in M: mycelia phase; Y: Yeast phase and C: protein common to the two fungal phases or protein with no differential expression. 8 ANOVA test - statistically significant differences are considered with p<0.05 (*); protein found at just one fungal phase (**). 9 Secretion prediction according to Signal P 3.0 server. The number corresponds to signal peptide probability (Score ≥0.5) (http://www.cbs.dtu.dk/services/SignalP/). 10 Secretion prediction according to Secretome P 2.0 server, the number corresponds to neural network that exceeded a value of 0.5 (NN-score ≥0.50) (http://www.cbs.dtu.dk/services/SecretomeP/). 11 Enzyme Classification recommended by Nomenclature Committee of the International Union of Biochemistry and Molecular Biology (NC-IUBMB). • adhesin-like proteins predicted by Faapred web server (http://bioinfo.icgeb.res.in/faap/query.html). (DOC) [file pone.0052470.s006.doc]

| **Spot Id^1^** | **General Information Number (NCBI) ^2^** | **Protein description** | **MM (kDa) theor/exp ^3^** | ***pI* (pH) theor/exp^4^** | **MS/MS Ion Score^5^** | **Matched peptides^6^** | **Isoform expression condition^7^** | **ANOVA (*p-*value)^8^** | **SignalP^9^ Score ≥ 0.5** | **SecretomeP^10^ Score ≥ 0.5** | **EC^11^** |  |
| --- | --- | --- | --- | --- | --- | --- | --- | --- | --- | --- | --- | --- |
| 116 | gi|295658863 | Cofilin/tropomyosin-type actin-binding family protein | 17.48/19.99 | 5.68/5.66 | 228 | 4 | M | ** | NO | NO |  |  |
| 129 | gi|295658863 | Cofilin/tropomyosin-type actin-binding family protein | 17.48/16.47 | 5.68/5.30 | 228 | 4 | C | 0.1581 | NO | NO |  |  |
| 10 | gi|295663469 | glycosyl hydrolase ● | 49.00/60.55 | 5.07/4.03 | 281 | 4 | C | 0.1350 | 0.996 | 0.8631 |  |  |
| 96 | gi|225678203 | NmrA-like family protein ● | 38.52/35.04 | 5.53/5.31 | 320 | 8 | C | 0.5624 | NO | 0.5898 |  |  |
| 56 | gi|295665666 | ribonucleoprotein ● | 35.09/52.37 | 7.88/4.24 | 133 | 4 | C | 0.6021 | NO | 0.9061 |  |  |
| 80 | gi|295665168 | TOS1 | 46.22/37.34 | 8.42/4.75 | 120 | 2 | C | 0.7772 | 0.999 | 0.9249 |  |  |
| 12 | gi|295659988 | 2,3-bisphosphoglycerate -independent phosphoglycerate mutase | 57.34/60.55 | 5.37/5.32 | 301 | 7 | M | ** | NO | 0.6367 | Isomerases |  |
| 77 | gi|295663891 | 2,5-diketo-D-gluconic acid reductase A | 32.04/49.73 | 6.67/5.62 | 346 | 9 | Y | ** | NO | NO | Oxidoreductases |  |
| 39 | gi|295666179 | 2-methylcitrate synthase | 51.51/48.88 | 9.02/8.41 | 269 | 8 | C | 0.1338 | NO | 0.5337 | Transferases |  |
| 40 | gi|295666179 | 2-methylcitrate synthase | 51.51/48.54 | 9.02/8.60 | 430 | 12 | M | 0.0008* | NO | 0.5337 | Transferases |  |
| 50 | gi|295666179 | 2-methylcitrate synthase | 51.51/44.21 | 9.02/8.27 | 54 | 2 | C | 0.4164 | NO | 0.5337 | Transferases |  |
| 51 | gi|295666179 | 2-methylcitrate synthase | 51.51/44.34 | 9.02/8.40 | 77 | 3 | C | 0.1891 | NO | 0.5337 | Transferases |  |
| 52 | gi|295666179 | 2-methylcitrate synthase | 51.51/45.15 | 9.02/8.61 | 348 | 7 | C | 0.0972 | NO | 0.5337 | Transferases |  |
| 53 | gi|295666179 | 2-methylcitrate synthase | 51.51/55.79 | 9.02/9.13 | 260 | 7 | Y | ** | NO | 0.5337 | Transferases |  |
| 68 | gi|295666179 | 2-methylcitrate synthase | 51.51/51.93 | 9.02/8.86 | 226 | 6 | Y | 0.0116* | NO | 0.5337 | Transferases |  |
| 69 | gi|295666179 | 2-methylcitrate synthase | 51.51/47.44 | 9.02/9.11 | 544 | 12 | Y | 0.0009* | NO | 0.5337 | Transferases |  |
| 70 | gi|295666179 | 2-methylcitrate synthase | 51.51/47.70 | 9.02/9.36 | 524 | 12 | Y | ** | NO | 0.5337 | Transferases |  |
| 101 | gi|295662074 | 3-hydroxybutyryl-CoA dehydrogenase | 34.36/38.53 | 8.41/6.91 | 209 | 7 | Y | ** | NO | NO | Oxidoreductases |  |
| 102 | gi|295663567 | 6-phosphogluconolactonase ● | 29.30/28.04 | 5.86/6.80 | 101 | 4 | C | 0.8172 | NO | 0.6288 | Hydrolases |  |
| 157 | gi|295663567 | 6-phosphogluconolactonase ● | 29.30/31.53 | 5.86/6.70 | 75 | 2 | M | ** | NO | 0.6288 | Hydrolases |  |
| 37 | gi|295659859 | acetyl-CoA acetyltransferase | 41.50/49.04 | 7.64/7.93 | 267 | 9 | C | 0.0623 | NO | 0.8393 | Transferases |  |
| 149 | gi|295659859 | acetyl-CoA acetyltransferase | 41.50/48.71 | 7.64/8.19 | 267 | 7 | M | ** | NO | 0.8393 | Transferases |  |
| 3 | gi|295664721 | aconitase | 79.19/82.62 | 6.49/7.47 | 299 | 10 | M | 0.0070* | NO | 0.6667 | Lyase |  |
| 4 | gi|295664721 | aconitase | 79.19/81.68 | 6.49/7.61 | 258 | 9 | M | 0.0007* | NO | 0.6667 | Lyase |  |
| 19 | gi|225684009 | aconitase | 85.06/72.66 | 7.28/7.20 | 153 | 6 | C | 0.3703 | NO | 0.5587 | Lyase |  |
| 8 | gi|295673184 | actin-interacting protein ● | 65.99/81.38 | 6.48/7.00 | 203 | 6 | Y | ** | NO | 0.9262 |  |  |
| 20 | gi|295673184 | actin-interacting protein ● | 65.99/65.93 | 6.48/7.39 | 144 | 5 | C | 0.1376 | NO | 0.9262 |  |  |
| 73 | gi|295674697 | adenosine kinase | 36.61/39.83 | 5.48/5.42 | 153 | 3 | M | 0.0182* | NO | NO | Transferases |  |
| 85 | gi|295674697 | adenosine kinase | 36.61/37.49 | 5.48/6.13 | 305 | 8 | C | 0.9409 | NO | NO | Transferases |  |
| 31 | gi|295665123 | aldehyde dehydrogenase | 54.55/50.05 | 5.87/6.14 | 376 | 10 | M | ** | NO | NO | Oxidoreductases |  |
| 9 | gi|295667902 | aminomethyltransferase ● | 53.16/72.66 | 9.59/7.20 | 279 | 8 | C | 0.2302 | NO | 0.6367 | Transferases |  |
| 136 | gi|295672500 | aminopeptidase | 55.17/59.06 | 5.42/6.17 | 185 | 6 | M | ** | 0.996 | 0.9401 | Hydrolases |  |
| 95 | gi|295667790 | beta-glucosidase | 34.05/35.29 | 6.96/4.77 | 340 | 5 | C | 0.2116 | 0.999 | 0.8279 | Hydrolases |  |
| 1 | gi|295664474 | cell division cycle protein | 90.56/82.24 | 4.98/4.52 | 299 | 8 | C | 0.2116 | NO | NO |  |  |
| 138 | gi|295671621 | choline dehydrogenase ● | 60.65/62.29 | 6.34/6.64 | 78 | 3 | M | ** | NO | NO | Oxidoreductases |  |
| 134 | gi|295659538 | Cobalamin-independent methionine synthase | 87.30/89.52 | 6.28/7.56 | 404 | 14 | M | ** | NO | NO | Transferases |  |
| 15 | gi|225680243 | cobalamin-independent synthase | 85.40/72.52 | 6.33/6.53 | 279 | 8 | Y | ** | NO | NO | Transferases |  |
| 120 | gi|295657286 | conserved hypothetical protein ● | 43.57/20.33 | 8.62/4.37 | 168 | 3 | C | 0.7950 | NO | 0.8050 |  |  |
| 74 | gi|295667926 | Conserved protein ● | 29.16/41.18 | 6.30/5.56 | 198 | 3 | C | 0.7060 | 0.972 | 0.9280 |  |  |
| 128 | gi|295670838 | nucleolar transport factor | 13.98/16.77 | 4.82/4.43 | 116 | 3 | Y | ** | NO | 0.9101 |  |  |
| 127 | gi|295660305 | cytochrome-c oxidase chain VI | 18.94/14.10 | 5.54/4.24 | 119 | 3 | Y | ** | 0.9240 | NO |  |  |
| 126 | gi|295660305 | cytochrome-c oxidase chain VI | 18.94/21.10 | 5.54/4.35 | 68 | 2 | Y | 0.0138* | 0.9240 | NO |  |  |
| 34 | gi|295668473 | dihydrolipoyl dehydrogenase | 56.06/58.32 | 8.27/7.38 | 191 | 8 | C | 0.9937 | 0.571 | NO | Oxidoreductases |  |
| 48 | gi|295668473 | dihydrolipoyl dehydrogenase | 56.06/46.82 | 8.27/7.32 | 677 | 11 | Y | 0.0194* | 0.5710 | NO | Oxidoreductases |  |
| 49 | gi|295668473 | dihydrolipoyl dehydrogenase | 56.06/55.58 | 8.27/7.52 | 310 | 9 | C | 0.0628 | 0.571 | NO | Oxidoreductases |  |
| 2 | gi|295660102 | dipeptidyl-peptidase | 86.54/105.25 | 7.99/7.26 | 88 | 3 | Y | ** | NO | NO | Hydrolases |  |
| 6 | gi|295666432 | dipeptidyl-peptidase | 78.80/67.06 | 5.49/4.59 | 248 | 9 | C | 0.2416 | NO | 0.9410 | Hydrolases |  |
| 18 | gi|295660102 | dipeptidyl-peptidase | 86.54/61.87 | 7.99/6.96 | 218 | 8 | C | 0.1757 | NO | NO | Hydrolases |  |
| 135 | gi|295666432 | dipeptidyl-peptidase | 66.30/66.30 | 5.49/4.77 | 115 | 5 | M | ** | NO | NO | Hydrolases |  |
| 21 | gi|295673162 | disulfide isomerase Pdi1 | 59.30/62.25 | 4.80/4.21 | 200 | 5 | Y | ** | 0.988 | 0.899 | Isomerases |  |
| 154 | gi|295673162 | disulfide isomerase Pdi1 | 59.30/27.74 | 4.80/3.88 | 290 | 4 | M | ** | 0.962 | 0.8994 | Isomerases |  |
| 151 | gi|295670457 | disulfide-isomerase tigA | 94.81/39.54 | 6.98/7.76 | 76 | 3 | M | ** | NO | NO | Isomerases |  |
| 64 | gi|295661300 | DNA damage checkpoint protein rad24 | 29.73/50.34 | 4.68/4.26 | 185 | 3 | Y | ** | NO | NO |  |  |
| 71 | gi|295661300 | DNA damage checkpoint protein rad24 | 29.73/48.27 | 4.68/4.80 | 101 | 2 | Y | ** | NO | NO |  |  |
| 83 | gi|295672736 | DNA damage checkpoint protein rad24 | 29.73/40.66 | 4.68/3.76 | 162 | 5 | C | 0.0693 | NO | NO |  |  |
| 94 | gi|295672736 | DNA damage checkpoint protein rad24 | 32.48/37.03 | 4.74/4.51 | 149 | 4 | C | 0.6128 | NO | NO |  |  |
| 140 | gi|295670934 | electron transfer flavoprotein-ubiquinone oxidoreductase | 71.41/59.46 | 7.59/6.54 | 160 | 5 | M | ** | NO | 0.8751 | Oxidoreductases |  |
| 150 | gi|295668925 | elongation factor 1-gamma 1 | 45.88/21.72 | 8.20/5.18 | 93 | 3 | M | ** | NO | 0.9067 |  |  |
| 88 | gi|295675019 | elongation factor 2 | 92.69/45.81 | 6.46/6.00 | 326 | 8 | M | ** | NO | NO |  |  |
| 27 | gi|295672732 | enolase ● | 43.85/48.71 | 8.93/9.83 | 316 | 7 | C | 0.0638 | NO | 0.5000 |  |  |
| 28 | gi|295672732 | enolase ● | 43.85/48.82 | 8.93/10.12 | 326 | 8 | C | 0.1495 | NO | 0.5000 | Lyase |  |
| 30 | gi|295672732 | enolase ● | 43.85/47.24 | 8.93/5.73 | 109 | 2 | M | 0.0011* | NO | 0.5491 | Lyase |  |
| 47 | gi|295672732 | enolase ● | 43.85/46.03 | 8.93/6.62 | 632 | 8 | C | 0.4405 | NO | 0.5491 | Lyase |  |
| 107 | gi|295662032 | enoyl-CoA hydratase | 32.11/30.18 | 8.89/8.74 | 317 | 7 | Y | ** | 0.699 | NO | Lyase |  |
| 156 | gi|295674311 | eukaryotic translation initiation factor 5A | 15.57/27.72 | 5.59/4.57 | 156 | 4 | M | 0.0007* | NO | NO |  |  |
| 119 | gi|295674311 | eukaryotic translation initiation factor 5A | 15.57/20.81 | 5.59/4.24 | 272 | 5 | Y | ** | NO | NO |  |  |
| 32 | gi|295668479 | formamidase | 46.14/50.91 | 6.10/6.96 | 202 | 8 | C | 0.1837 | NO | NO |  |  |
| 33 | gi|295668479 | formamidase | 46.14/50.11 | 6.10/7.07 | 149 | 6 | C | 0.1551 | NO | NO | Hydrolases |  |
| 35 | gi|295668479 | formamidase | 46.14/53.09 | 6.10/7.42 | 218 | 7 | C | 0.1034 | NO | NO | Hydrolases |  |
| 36 | gi|295668479 | formamidase | 46.14/49.60 | 6.10/7.66 | 91 | 3 | C | 0.0663 | NO | NO | Hydrolases |  |
| 145 | gi|295668479 | formamidase | 46.14/52.14 | 6.10/7.51 | 91 | 3 | M | ** | NO | NO | Hydrolases |  |
| 63 | gi|295671120 | fructose-bisphosphate aldolase | 39.72/39.75 | 6.09/7.08 | 458 | 6 | C | 0.1520 | NO | 0.6628 | Lyase |  |
| 81 | gi|295671120 | fructose-bisphosphate aldolase | 39.72/37.96 | 6.09/7.10 | 154 | 5 | C | 0.4368 | NO | 0.6628 | Lyase |  |
| 89 | gi|295671120 | fructose-bisphosphate aldolase | 39.72/43.17 | 6.09/6.72 | 154 | 5 | Y | ** | NO | 0.6628 | Lyase |  |
| 90 | gi|295671120 | fructose-bisphosphate aldolase | 39.72/33.61 | 6.09/7.49 | 670 | 5 | Y | 0.012* | NO | 0.6628 | Lyase |  |
| 91 | gi|295671120 | fructose-bisphosphate aldolase | 39.72/34.10 | 6.09/7.78 | 555 | 9 | C | 0.8995 | NO | 0.6628 | Lyase |  |
| 60 | gi|295658698 | fumarylacetoacetase | 46.75/52.33 | 5.95/6.22 | 278 | 7 | C | 0.0862 | NO | 0.7492 | Hydrolases |  |
| 61 | gi|295658698 | fumarylacetoacetase | 46.75/41.70 | 5.95/6.71 | 283 | 8 | C | 0.0701 | NO | 0.7492 | Hydrolases |  |
| 62 | gi|295658698 | fumarylacetoacetase | 46.75/42.75 | 5.95/7.14 | 132 | 4 | C | 0.3835 | NO | 0.7492 | Hydrolases |  |
| 79 | gi|295658698 | fumarylacetoacetase | 46.75/39.50 | 5.95/6.56 | 153 | 5 | C | 0.7047 | NO | 0.7492 | Hydrolases |  |
| 111 | gi|295667597 | G4 quadruplex nucleic acid binding protein | 59.55/25.38 | 9.23/6.27 | 86 | 2 | Y | ** | NO | 0.9401 |  |  |
| 118 | gi|295660961 | gamma-glutamyltranspeptidase | 64.14/24.67 | 6.16/7.98 | 95 | 2 | Y | ** | NO | NO | Transferases |  |
| 23 | gi|295657201 | glutamate carboxypeptidase | 64.61/54.06 | 6.23/5.41 | 376 | 9 | M | 0.0064* | NO | 0.7370 | Hydrolases |  |
| 44 | gi|295657201 | glutamate carboxypeptidase | 64.61/56.27 | 6.23/5.63 | 180 | 6 | C | 0.2385 | NO | 0.7370 | Hydrolases |  |
| 25 | gi|295664022 | glutathione reductase | 51.95/52.08 | 6.74/8.07 | 192 | 5 | M | 0.0248* | NO | NO | Oxidoreductases |  |
| 26 | gi|295664022 | glutathione reductase | 51.95/49.94 | 6.74/9.06 | 114 | 5 | M | 0.0279* | NO | NO | Oxidoreductases |  |
| 38 | gi|295664022 | glutathione reductase | 51.95/48.99 | 6.74/8.08 | 165 | 5 | C | 0.7462 | NO | NO | Oxidoreductases |  |
| 84 | gi|295664022 | glutathione reductase | 51.95/42.96 | 6.74/4.14 | 81 | 3 | C | 0.3052 | NO | NO | Oxidoreductases |  |
| 67 | gi|295667577 | glutathione S-transferase Gst3 ● | 45.30/41.35 | 8.29/8.26 | 216 | 5 | Y | ** | NO | 0.5119 | Transferases |  |
| 82 | gi|295658119 | glyceraldehyde-3-phosphate dehydrogenase ● | 36.61/33.92 | 8.26/10.18 | 93 | 2 | C | 0.4655 | NO | 0.9120 | Oxidoreductases |  |
| 93 | gi|295658119 | glyceraldehyde-3-phosphate dehydrogenase ● | 36.61/34.17 | 8.26/8.49 | 444 | 12 | C | 0.4664 | NO | 0.9120 | Oxidoreductases |  |
| 17 | gi|295658865 | heat shock protein 60 ● | 62.26/65.81 | 5.51/6.83 | 174 | 7 | M | 0.0391* | NO | NO |  |  |
| 29 | gi|295658865 | heat shock protein 60 ● | 62.26/59.55 | 5.51/4.23 | 461 | 11 | Y | ** | NO | NO |  |  |
| 41 | gi|295658865 | heat shock protein 60 ● | 62.26/56.49 | 5.51/4.68 | 345 | 8 | Y | ** | NO | NO |  |  |
| 42 | gi|295658865 | heat shock protein 60 ● | 62.26/56.22 | 5.51/4.97 | 428 | 9 | Y | ** | NO | NO |  |  |
| 137 | gi|295658865 | heat shock protein 60 ● | 62.26/59.66 | 5.51/6.40 | 115 | 3 | M | ** | NO | NO |  |  |
| 5 | gi|295659787 | heat shock protein Hsp88 | 80.68/69.89 | 4.92/4.52 | 272 | 6 | C | 0.3645 | NO | NO |  |  |
| 54 | gi|295659837 | heat shock protein SSB1 | 60.69/51.55 | 5.47/3.91 | 286 | 6 | C | 0.07791 | NO | 0.8618 |  |  |
| 7 | gi|295671569 | heat shock protein SSC1 | 73.82/64.66 | 5.92/5.14 | 342 | 8 | M | 0.0344* | NO | 0.6791 |  |  |
| 13 | gi|295671569 | heat shock protein SSC1 | 73.82/65.50 | 5.92/5.36 | 468 | 11 | C | 0.0789 | NO | 0.6791 |  |  |
| 14 | gi|295671569 | heat shock protein SSC1 | 73.82/59.19 | 5.92/5.82 | 468 | 11 | Y | 0.0100* | NO | 0.6791 |  |  |
| 43 | gi|295671569 | heat shock protein SSC1 | 73.82/45.10 | 5.92/5.56 | 302 | 9 | M | 0.0175* | NO | 0.6791 |  |  |
| 45 | gi|295671569 | heat shock protein SSC1 | 73.82/44.90 | 5.92/5.93 | 546 | 12 | M | 0.0006* | NO | 0.6791 |  |  |
| 46 | gi|295671569 | heat shock protein SSC1 | 73.82/44.44 | 5.92/6.37 | 375 | 10 | M | 0.0319* | NO | 0.6791 |  |  |
| 59 | gi|295671569 | heat shock protein SSC1 | 73.82/55.01 | 5.92/5.67 | 403 | 10 | Y | ** | NO | 0.6791 |  |  |
| 148 | gi|295671569 | heat shock protein SSC1 | 73.82/44.25 | 5.92/8.00 | 197 | 5 | M | ** | NO | 0.6791 |  |  |
| 141 | gi|295664250 | histidine biosynthesis trifunctional protein | 93.53/52.91 | 5.79/7.07 | 490 | 12 | M | ** | NO | 0.6345 | Hydrolase and Oxidoreductase |  |
| 11 | gi|295659116 | hsp70-like protein | 70.91/58.73 | 5.08/5.20 | 431 | 12 | Y | 0.0161* | NO | 0.5000 |  |  |
| 22 | gi|295659116 | hsp70-like protein | 70.91/57.28 | 5.08/4.66 | 554 | 13 | C | 0.0548 | NO | 0.5000 |  |  |
| 57 | gi|295659116 | hsp70-like protein | 70.91/53.37 | 5.08/4.38 | 462 | 8 | Y | ** | NO | 0.5000 |  |  |
| 72 | gi|295659116 | hsp70-like protein | 70.91/41.48 | 5.08/5.33 | 315 | 10 | C | 0.0760 | NO | 0.5000 |  |  |
| 76 | gi|295659116 | hsp70-like protein | 70.91/41.44 | 5.08/6.13 | 613 | 13 | Y | 0.0144* | NO | 0.5000 |  |  |
| 86 | gi|295659116 | hsp70-like protein | 70.91/40.00 | 5.08/6.28 | 634 | 12 | Y | 0.0245* | NO | 0.5000 |  |  |
| 147 | gi|295673716 | hsp70-like protein | 68.85/47.24 | 5.39/5.73 | 225 | 8 | M | ** | 0.983 | NO |  |  |
| 108 | gi|295665077 | Hsp90 binding co-chaperone (Sba1) | 21.30/29.90 | 4.23/4.08 | 212 | 3 | C | 0.1321 | NO | NO |  |  |
| 115 | gi|295665077 | Hsp90 binding co-chaperone (Sba1) | 21.30/21.77 | 4.23/4.50 | 126 | 3 | Y | 0.0392* | NO | NO |  |  |
| 121 | gi|295658312 | L-PSP endoribonuclease family protein | 18.72/19.31 | 8.96/5.77 | 118 | 3 | M | ** | NO | NO |  |  |
| 92 | gi|295673937 | malate dehydrogenase | 36.02/32.30 | 8.99/8.25 | 186 | 4 | C | 0.0834 | 0.764 | 0.8163 | Oxidoreductases |  |
| 87 | gi|295662360 | mannitol-1-phosphate 5-dehydrogenase | 43.11/36.30 | 5.66/6.49 | 292 | 9 | C | 0.4364 | NO | NO | Oxidoreductases |  |
| 99 | gi|295662360 | mannitol-1-phosphate 5-dehydrogenase | 43.11/45.42 | 5.66/5.37 | 376 | 10 | Y | ** | NO | NO | Oxidoreductases |  |
| 75 | gi|295662360 | mannitol-1-phosphate 5-dehydrogenase | 43.11/40.97 | 5.66/5.96 | 197 | 4 | C | 0.1021 | NO | NO | Oxidoreductases |  |
| 142 | gi|295661139 | methylmalonate-semialdehyde dehydrogenase | 63.10/57.87 | 8.99/7.30 | 111 | 5 | M | ** | NO | 0.8078 | Oxidoreductases |  |
| 143 | gi|295661139 | methylmalonate-semialdehyde dehydrogenase | 63.10/57.60 | 8.99/7.62 | 142 | 6 | M | ** | NO | 0.8078 | Oxidoreductases |  |
| 155 | gi|295658437 | mitochondrial-processing peptidase subunit alpha ● | 64.22/27.33 | 7.02/4.47 | 78 | 3 | M | ** | NO | 0.6031 | Hydrolases |  |
| 106 | gi|295668188 | nuclear movement protein nudC | 22.39/29.14 | 5.64/5.52 | 138 | 5 | Y | ** | NO | 0.7668 |  |  |
| 103 | gi|295665468 | nucleic acid-binding protein | 30.40/28.22 | 9.40/7.63 | 167 | 4 | C | 0.9758 | 0.982 | 0.5915 |  |  |
| 113 | gi|295665468 | nucleic acid-binding protein | 30.40/24.64 | 9.40/6.74 | 132 | 4 | Y | 0.0103* | 0.982 | 0.5915 |  |  |
| 130 | gi|295666938 | nucleoside diphosphate kinase | 16.87/14.54 | 6.84/6.44 | 209 | 8 | C | 0.1512 | NO | NO | Transferases |  |
| 133 | gi|295666938 | nucleoside diphosphate kinase | 16.87/15.77 | 6.84/7.64 | 249 | 7 | C | 0.5725 | NO | NO | Transferases |  |
| 65 | gi|295658947 | O-acetylhomoserine (thiol)-lyase ● | 47.12/51.98 | 6.24/7.19 | 443 | 10 | C | 0.9268 | NO | 0.7930 | Transferases |  |
| 122 | gi|295663907 | peptidyl-prolyl cis-trans isomerase A2 | 27.73/18.69 | 9.41/7.11 | 75 | 3 | C | 0.4941 | 0.975 | 0.8963 | Isomerases |  |
| 123 | gi|295672668 | peptidyl-prolyl cis-trans isomerase B | 22.81/18.12 | 7.88/8.18 | 209 | 4 | C | 0.1613 | 0.928 | NO | Isomerases |  |
| 159 | gi|295672668 | peptidyl-prolyl cis-trans isomerase B | 22.81/19.37 | 7.88/7.99 | 261 | 7 | M | ** | 0.928 | NO | Isomerases |  |
| 132 | gi|295662699 | peptidyl-prolyl cis-trans isomerase cypE | 17.52/15.18 | 6.07/6.66 | 134 | 3 | C | 0.2723 | NO | 0.8712 | Isomerases |  |
| 58 | gi|295668481 | peptidyl-prolyl cis-trans isomerase D | 41.36/51.84 | 5.36/5.26 | 215 | 6 | Y | ** | NO | 0.8640 | Isomerases |  |
| 158 | gi|295668481 | peptidyl-prolyl cis-trans isomerase D | 41.36/21.87 | 5.36/5.41 | 235 | 5 | M | ** | NO | 0.8640 | Isomerases |  |
| 124 | gi|295672447 | peptidyl-prolyl cis-trans isomerase H | 20.02/18.85 | 8.80/8.74 | 137 | 3 | C | 0.0634 | NO | 0.7266 | Isomerases |  |
| 125 | gi|295672447 | peptidyl-prolyl cis-trans isomerase H | 20.02/16.78 | 8.80/9.20 | 199 | 6 | C | 0.1824 | NO | 0.7266 | Isomerases |  |
| 160 | gi|295672447 | peptidyl-prolyl cis-trans isomerase H | 20.02/18.00 | 8.80/9.20 | 130 | 3 | M | ** | NO | 0.7266 | Isomerases |  |
| 24 | gi|225681400 | peroxisomal catalase | 57.65/55.74 | 6.42/7.56 | 255 | 7 | C | 0.4301 | NO | 0.9389 | Oxidoreductases |  |
| 144 | gi|225681400 | peroxisomal catalase | 57.65/56.12 | 6.42/7.67 | 255 | 7 | M | ** | NO | 0.9389 | Oxidoreductases |  |
| 66 | gi|295669690 | phosphoglycerate kinase | 45.31/42.81 | 6.48/7.57 | 383 | 11 | C | 0.3639 | NO | 0.6626 | Transferases |  |
| 139 | gi|295668873 | phosphoribosylamine-glycine ligase ● | 85.25/52.31 | 5.72/6.05 | 117 | 6 | M | ** | NO | NO | Ligases |  |
| 98 | gi|295672926 | proteasome component PRE4 | 29.28/30.12 | 6.17/6.80 | 132 | 3 | M | 0.0057* | NO | 0.5976 | Hydrolases |  |
| 112 | gi|295672926 | proteasome component PRE4 | 29.28/28.43 | 6.17/6.88 | 68 | 3 | Y | 0.0073* | NO | NO | Hydrolases |  |
| 16 | gi|295662174 | pyruvate kinase | 59.48/71.58 | 6.31/6.71 | 139 | 6 | Y | ** | NO | NO | Transferases |  |
| 146 | gi|295659992 | serine hydroxymethyltransferase | 52.42/51.37 | 8.29/8.45 | 111 | 4 | M | ** | NO | NO | Transferases |  |
| 97 | gi|225683737 | spermidine synthase | 33.71/33.64 | 5.33/5.29 | 200 | 4 | C | 0.0864 | NO | NO | Transferases |  |
| 104 | gi|295669402 | Mn superoxide dismutase | 24.81/26.47 | 8.91/8.50 | 222 | 5 | C | 0.3780 | NO | 0.8823 | Oxidoreductases |  |
| 55 | gi|295666684 | Cu - Zn superoxide dismutase ● | 15.97/50.51 | 5.92/4.11 | 132 | 4 | Y | ** | NO | 0.9184 | Oxidoreductases |  |
| 131 | gi|295666684 | Cu - Zn superoxide dismutase ● | 15.97/14.60 | 5.92/6.47 | 135 | 4 | C | 0.0503 | NO | 0.9185 | Oxidoreductases |  |
| 109 | gi|295656848 | TCTP family protein | 20.28/28.80 | 4.75/4.40 | 164 | 3 | M | ** | NO | NO |  |  |
| 78 | gi|295661107 | thioredoxin reductase ● | 38.18/37.38 | 5.51/6.49 | 405 | 7 | M | ** | NO | 0.966 | Oxidoreductases |  |
| 100 | gi|295659831 | thioredoxin-like protein ● | 23.61/29.39 | 6.21/6.60 | 116 | 4 | M | 0.0087* | NO | 0.8876 |  |  |
| 114 | gi|295659831 | Thioredoxin-like protein ● | 23.61/23.74 | 6.21/6.71 | 220 | 7 | C | 0.0862 | NO | 0.8876 |  |  |
| 117 | gi|295659831 | Thioredoxin-like protein ● | 23.61/19.67 | 6.21/7.20 | 104 | 3 | Y | 0.0046* | NO | 0.8876 |  |  |
| 152 | gi|295666688 | transaldolase | 35.75/40.04 | 6.47/8.08 | 272 | 11 | M | ** | NO | NO | Transferases |  |
| 153 | gi|295666688 | transaldolase | 35.75/40.71 | 6.47/8.11 | 276 | 12 | M | ** | NO | NO | Transferases |  |
| 110 | gi|295670663 | triosephosphate isomerase ● | 27.15/27.86 | 5.39/5.35 | 346 | 7 | M | 0,0132* | NO | NO | Isomerases |  |
| 105 | gi|295662829 | vesicular-fusion protein SEC17 | 32.78/30.63 | 5.44/4.98 | 377 | 8 | Y | ** | NO | NO |  |  |
