# Supplementary material for: Analysis of the Secretomes of Paracoccidioides Mycelia and Yeast Cells
Source: PLoS One. 2012 Dec 18;7(12):e52470. doi: 10.1371/journal.pone.0052470 (PMC3525554; doi:10.1371/journal.pone.0052470)
Supplement: Table S2 — Exclusive proteins secreted by Paracoccidioides Pb 01 yeast and mycelia. 1 Spots numbers indicated in Figure 2. 2 NCBI database general information number (http://www.ncbi.nlm.nih.gov/). 3 Number of identified isoforms of protein in each Paracoccidioides, Pb01 phase secretome. 4 The average of amount of values of abundances of all identified isoforms. 5 Protein Expression in M: mycelia phase; Y: Yeast phase. 6 Secretion prediction according to Signal P 3.0 server, the number corresponds to signal peptide probability (http://www.cbs.dtu.dk/services/SignalP/). 7 Secretion prediction according to Secretome P 2.0 server, the number corresponds to neural network that exceeded a value of 0.5 (NN-score ≥0.50) (http://www.cbs.dtu.dk/services/SecretomeP/). (DOC) [file pone.0052470.s007.doc]

|  |  |  |  |  |  |  |  |
| --- | --- | --- | --- | --- | --- | --- | --- |
| **Spot number^1^** | **General Information number (NCBI) ^2^** | **Protein description** | **Number of isoforms in mycelia ^3^** | **Amount of isoform abundances ^4^** | **Protein expression condition ^5^** | **Signal P Score ≥ 0.5^6^** | **SecretomeP Score ≥ 0.5^7^** |
|
| **1. CELL RESCUE, DEFENSE and VIRULENCE** | | |  |  |  |  |  |
| **1.1. Stress response** | |  |  |  |  |  |  |
| 67 | gi|295667577 | glutathione S-transferase Gst3 | 1 | 2.44 | Y | NO | 0.5119 |
|  |  |  |  |  |  |  |  |
| **1.2. Detoxification** | |  |  |  |  |  |  |
| 151 | gi|295670457 | disulfide-isomerase tigA | 1 | 0.07 | M | NO | NO |
| 78 | gi|295661107 | thioredoxin reductase | 1 | 1.47 | M | NO | 0.966 |
| 109 | gi|295656848 | TCTP family protein | 1 | 1.08 | M | NO | NO |
|  |  |  |  |  |  |  |  |
| **2. METABOLISM** | | |  |  |  |  |  |
| **2.1. Amino Acid Metabolism** | | |  |  |  |  |  |
| 138 | gi|295671621 | choline dehydrogenase | 1 | 0.09 | M | NO | NO |
| 134 | gi|295659538 | Cobalamin-independent methionine synthase | 1 | 0.04 | M | NO | NO |
| 141 | gi|295664250 | histidine biosynthesis trifunctional protein | 1 | 0.32 | M | NO | 0.6345 |
| 142 | gi|295661139 | methylmalonate-semialdehyde dehydrogenase | 2 | 0.41 | M | NO | 0.8078 |
| 146 | gi|295659992 | serine hydroxymethyltransferase | 1 | 0,15 | M | NO | NO |
| 15 | gi|225680243 | Cobalamin-independent methionine synthase | 1 | 0.04 | Y | NO | NO |
|  |  |  |  |  |  |  |  |
| **2.2. Secundary Metabolism** | | |  |  |  |  |  |
| 77 | gi|295663891 | 2,5-diketo-D-gluconic acid reductase A | 1 | 0.09 | Y | NO | NO |
|  |  |  |  |  |  |  |  |
| **2.3. Phosphate Metabolism** | | |  |  |  |  |  |
| 139 | gi|295668873 | phosphoribosylamine-glycine ligase | 1 | 0.14 | M | NO | NO |
|  |  |  |  |  |  |  |  |
| **2.4. C-Compound and Carbohydrate Metabolism** | | |  |  |  |  |  |
| 31 | gi|295665123 | aldehyde dehydrogenase | 1 | 0.14 | M | NO | NO |
|  |  |  |  |  |  |  |  |
| **3. NERGY** |  |  |  |  |  |  |  |
| **3.1. Glycolysis and Gluconeogenesis** | | |  |  |  |  |  |
| 16 | gi|295662174 | pyruvate kinase | 1 | 0.09 | Y | NO | NO |
| 12 | gi|295659988 | 2,3-bisphosphoglycerate -independent phosphoglycerate mutase | 1 | 1.20 | M | NO | 0.6367 |
|  |  |  |  |  |  |  |  |
| **3.2. Oxidation of fatty acids** | | |  |  |  |  |  |
| 140 | gi|295670934 | electron transfer flavoprotein-ubiquinone oxidoreductase | 1 | 0.10 | M | NO | 0.8751 |
| 107 | gi|295662032 | enoyl-CoA hydratase | 1 | 0.89 | Y | 0.699 | NO |
| 101 | gi|295662074 | 3-hydroxybutyryl-CoA dehydrogenase | 1 | 1.79 | Y | NO | NO |
|  |  |  |  |  |  |  |  |
| **3.4. Pentose-phophate Pathway** | | |  |  |  |  |  |
| 152 | gi|295666688 | transaldolase | 2 | 0.53 | M | NO | NO |
|  |  |  |  |  |  |  |  |
| **4. CELL CYCLE AND DNA PROCESSING** | | |  |  |  |  |  |
| 64 | gi|295661300 | DNA damage checkpoint protein rad24 | 2 | 0.85 | Y | NO | NO |
| 106 | gi|295668188 | nuclear movement protein nudC | 1 | 0.24 | Y | NO | 0.7668 |
| 111 | gi|295667597 | G4 quadruplex nucleic acid binding protein | 1 | 1.96 | Y | NO | 0.9401 |
|  |  |  |  |  |  |  |  |
| **5. PROTEIN FATE (folding, modification, destination)** | | |  |  |  |  |  |
| 136 | gi|295672500 | aminopeptidase | 1 | 0.33 | M | 0.996 | 0.9401 |
| 155 | gi|295658437 | mitochondrial-processing peptidase subunit alpha | 1 | 0.09 | M | NO | 0.6031 |
| 118 | gi|295660961 | gamma-glutamyltranspeptidase | 1 | 1.26 | Y | NO | NO |
|  |  |  |  |  |  |  |  |
| **6. PROTEIN SYNTHESIS** | |  |  |  |  |  |  |
| 150 | gi|295668925 | elongation factor 1-gamma 1 | 1 | 0.19 | M | NO | 0.9067 |
| 88 | gi|295675019 | elongation factor 2 | 1 | 2.54 | M | NO | NO |
|  |  |  |  |  |  |  |  |
| **7. CELLULAR TRANSPORT, TRANSPORT FACILITIES AND TRANSPORT ROUTES** | | | | | | | |
| 127 | gi|295660305 | cytochrome-c oxidase chain VI | 1 | 1.49 | Y | 0.9240 | NO |
| 105 | gi|295662829 | vesicular-fusion protein SEC17 | 1 | 1.38 | Y | NO | NO |
|  |  |  |  |  |  |  |  |
| **8. UNCLASSIFIED PROTEINS** | | |  |  |  |  |  |
| 121 | gi|295658312 | L-PSP endoribonuclease family protein | 1 | 2.02 | M | NO | NO |
| 128 | gi|295670838 | conserved protein | 1 | 0.85 | Y | NO | 0.9101 |
|  | | | | | | | |
