# Supplementary material for: Analysis of the Secretomes of Paracoccidioides Mycelia and Yeast Cells
Source: PLoS One. 2012 Dec 18;7(12):e52470. doi: 10.1371/journal.pone.0052470 (PMC3525554; doi:10.1371/journal.pone.0052470)
Supplement: Table S3 — Predicted post-translational modifications of identified protein isoforms in mycelia and yeast secretomes. 1 Protein Expression in M: mycelia phase; Y: Yeast phase and C: protein common or protein with no differential expression. 2 PTM – Post translational modifications: acetyl (k) – lysine acetylation; phospo (S-T-Y) – serine, threonine and tyrosine phosphorylation. 3 Values returned by MASCOT search tool using none or specific variable modifications. (DOC) [file pone.0052470.s008.doc]

| **Protein descritpion** | **General Information Number (NCBI)** | **Spot number** | **Preferential expression^1^** | **Theo. Mr (kDa)/*pI*** | **Expt. Mr (kDa)/*pI*** | **PTM^2^** | **Sequence coverage%** | **Nº of mass values matched^3^** |
| --- | --- | --- | --- | --- | --- | --- | --- | --- |
| hsp70-like protein | gi|295659116 | 76 | Y | 70.91/5.08 | 41.44/6.13 | - | 41 | 30 |
|  |  |  |  |  |  | acetyl (k) | 45 | 34 |
|  |  | 86 | Y | 70.91/5.08 | 40.00/6.28 | - | 44 | 33 |
|  |  |  |  |  |  | acetyl (k) | 51 | 46 |
|  |  | 57 | Y | 70.91/5.08 | 53.37/4.38 | - | 50 | 32 |
|  |  |  |  |  |  | acetyl (k) | 56 | 40 |
|  |  |  |  |  |  | phospo (ST) | 52 | 39 |
| aconitase | gi|295664721 | 3 | M | 79.19/6.49 | 82.62/4.52 | - | 51 | 41 |
|  |  |  |  |  |  | phospho (ST) | 52 | 46 |
|  |  |  |  |  |  | phospho (Y) | 52 | 43 |
| mannitol-1-phosphate 5-dehydrogenase | gi|295662360 | 99 | Y | 43.11/5.66 | 45.42/5.37 | - | 79 | 29 |
|  |  |  |  |  |  | phospho (Y) | 79 | 30 |
| methylmalonate-semialdehyde dehydrogenase | gi|295661139 | 142 | M | 63.1/8.99 | 57.6/7.62 | - | 59 | 30 |
|  |  |  |  |  |  | acetyl (k) | 63 | 41 |
| nucleoside diphosphate kinase | gi|295666938 | 130 | C | 16.87/6.84 | 14.54/6.44 | - | 62 | 11 |
|  |  |  |  |  |  | acetyl (k) | 62 | 12 |
| peptidyl-prolyl cis-trans isomerase B | gi|295672668 | 159 | M | 22.81/7.88 | 19.37/7.99 | - | 69 | 16 |
|  |  |  |  |  |  | acet (K) | 71 | 21 |
|  |  |  |  |  |  | phospho (Y) | 69 | 17 |
| peptidyl-prolyl cis-trans isomerase D | gi|295668481 | 58 | Y | 41.36/5.36 | 51.84/5.26 | - | 47 | 18 |
|  |  |  |  |  |  | acetyl (k) | 54 | 21 |
|  |  |  |  |  |  | phospho (ST) | 48 | 20 |
| peptidyl-prolyl cis-trans isomerase H | gi|295672447 | 160 | M | 20.02/8.80 | 18.00/9.20 | - | 73 | 20 |
|  |  |  |  |  |  | phospho (ST) | 73 | 23 |
|  |  | 124 | C | 20.02/8.80 | 18.85/8.74 | - | 46 | 14 |
|  |  |  |  |  |  | acetyl (k) | 51 | 16 |
| 2-methylcitrate synthase | gi|295666179 | 40 | M | 51.51/ 9.02 | 48.54/8.60 | - | 59 | 30 |
|  |  |  |  |  |  | phospho (ST) | 62 | 33 |
|  |  |  |  |  |  |  |  |  |
|  | gi\|295666179 | 50 | C | 51.51/ 9.02 | 44.21/8.27 | - | 43 | 24 |
|  |  |  |  |  |  | phospho (ST) | 48 | 30 |
|  |  |  |  |  |  | Acet (K) | 50 | 30 |
|  | gi\|295666179 | 69 | Y | 51.51/ 9.02 | 47.44/9.11 | - | 45 | 27 |
|  |  |  |  |  |  | phospho (ST) | 51 | 33 |
|  |  |  |  |  |  | acetyl (k) | 48 | 32 |
|  | gi\|295666179 | 68 | Y | 51.51/ 9.02 | 51.93/8.86 | - | 48 | 26 |
|  |  |  |  |  |  | phospho (ST) | 48 | 29 |
|  |  |  |  |  |  | acetyl (k) | 53 | 31 |
|  | gi\|295666179 | 53 | Y | 51.51/ 9.02 | 55.79/9.13 | - | 52 | 27 |
|  |  |  |  |  |  | phospho (ST) | 61 | 39 |
|  |  |  |  |  |  | acetyl (k) | 60 | 32 |
|  | gi\|295666179 | 70 | Y | 51.51/ 9.02 | 47.70/9.36 | - | 60 | 29 |
|  |  |  |  |  |  | phospho (ST) | 57 | 30 |
|  |  |  |  |  |  | acetyl (k) | 59 | 29 |
|  | gi\|295666179 | 52 | Y | 51.51/ 9.02 | 45.15/8.61 | - | 38 | 21 |
|  |  |  |  |  |  | phospho (ST) | 51 | 31 |
|  |  |  |  |  |  | acetyl (k) | 46 | 23 |
|  | gi\|295666179 | 39 | C | 51.51/ 9.02 | 48.88/8.41 | - | 54 | 28 |
|  |  |  |  |  |  | phospho (ST) | 62 | 33 |
|  |  |  |  |  |  | acetyl (k) | 59 | 30 |
| 6-phosphoglunconolactonase | gi|295663567 | 157 | M | 29.30/5.86 | 31.53/6.70 | - | 73 | 22 |
|  |  |  |  |  |  | phospho (ST) | 74 | 31 |
|  |  |  |  |  |  | acetyl (k) | 73 | 26 |
|  |  | 102 | C | 29.30/5.86 | 28.04/6.80 | - | 68 | 25 |
|  |  |  |  |  |  | phospho (ST) | 74 | 48 |
|  |  |  |  |  |  | acetyl (k) | 70 | 32 |
| adenosine kinase | gi|295674697 | 73 | M | 36.61/5.48 | 39.83/5.42 | - | 54 | 22 |
|  |  |  |  |  |  | phospho (ST) | 55 | 24 |
|  |  |  |  |  |  | acetyl (k) | 57 | 25 |
|  |  | 85 | C | 36.61/5.48 | 37.49/6.13 | - | 53 | 18 |
| thioredoxin-like protein | gi|195659831 | 100 | M | 23.61/6.21 | 29.39/6.60 | - | 88 | 15 |
|  |  |  |  |  |  | phospho (ST) | 92 | 20 |
|  |  |  |  |  |  | acetyl (k) |  | 17 |
| dihydrolipoyl dehydrogenase | gi|295668473 | 48 | Y | 56.06/8.27 | 46.82/7.32 | - | 57 | 33 |
|  |  |  |  |  |  | acetyl (k) | 60 | 37 |
| dipeptidil peptidase | gi|295666432 | 135 | M | 66.30/5.49 | 66.30/4.77 | - | 52 | 40 |
| dipeptidil peptidase | gi|295660102 | 18 | C | 86.54/7.99 | 61.87/6.96 | - | 67 | 56 |
|  |  |  |  |  |  | phospho (ST) | 70 | 63 |
|  |  |  |  |  |  | acetyl (k) | 71 | 71 |
| disulfide isomerase | gi295673162 | 154 | M | 59.30/4.80 | 27.74/3.88 | - | 62 | 34 |
|  |  |  |  |  |  | acetyl (k) | 71 | 53 |
| DNA damage checkpoint protein rad24 | gi295661300 | 64 | Y | 29.73/4.68 | 50.34/4.26 | - | 73 | 26 |
|  |  |  |  |  |  | acetyl (k) | 76 | 33 |
| enolase | gi295672732 | 47 |  |  |  | - | 69 | 30 |
|  |  |  |  |  |  | phospho (ST) | 71 | 37 |
| fructose-biphosphate aldolase | gi295671120 | 91 | C | 39.72/6.09 | 34.10/7.78 | - | 61 | 18 |
|  |  |  |  |  |  | acetyl (k) | 61 | 23 |
|  |  | 89 | Y | 39.72/6.09 | 43.17/6.72 | - | 65 | 22 |
|  |  |  |  |  |  | acetyl (k) | 67 | 28 |
|  |  | 63 | C | 39.72/6.09 | 34.10/7.78 | - | 65 | 25 |
|  |  |  |  |  |  | acetyl (k) | 66 | 26 |
| fumarylacetoacetase | gi295658698 | 61 | C | 46.75/5.95 | 41.70/6.71 | - | 53 | 22 |
|  |  |  |  |  |  | Phosphor(S/T) | 59 | 27 |
| glutamate carboxypeptidase | gi|295657201 | 44 | C | 64.61/6.23 | 56.27/5.63 | - | 44 | 27 |
|  |  |  |  |  |  | phospho (ST) | 56 | 33 |
| glyceraldehyde-3-phosphate dehydrogenase | gi|295658119 | 93 | C | 36.61/8.26 | 34.17/8.49 | - | 87 | 26 |
|  |  |  |  |  |  | acetyl (k) | 92 | 31 |
| heat shock protein 60 | gi295658865 | 137 | M | 62.26/5.51 | 59.66/6.40 | - | 61 | 35 |
|  |  |  |  |  |  | phospho (ST) | 72 | 46 |
|  |  |  |  |  |  | acetyl (k) | 72 | 46 |
|  |  | 17 | M | 62.26/5.51 | 65.81/6.83 | - | 67 | 46 |
|  |  |  |  |  |  | phospho (ST) | 81 | 59 |
|  |  | 29 | Y | 62.26/5.51 | 59.55/4.23 | - | 79 | 49 |
|  |  |  |  |  |  | phospho (ST) | 88 | 58 |
|  |  |  |  |  |  |  | 80 | 55 |
|  |  | 42 | Y | 62.26/5.51 | 56.22/4.97 | - | 87 | 63 |
|  |  |  |  |  |  | phospho (ST) | 92 | 82 |
|  |  |  |  |  |  | acetyl (k) | 88 | 74 |
|  |  | 41 | Y | 62.26/5.51 | 56.49/4.68 | - | 61 | 43 |
|  |  |  |  |  |  | phospho (ST) | 62 | 46 |
|  |  |  |  |  |  |  | 64 | 52 |
| heat shock protein SSC1 | gi295671569 | 7 | M | 73.82/5.92 | 64.66/5.14 | - | 49 | 35 |
|  |  |  |  |  |  | phospho (ST) | 52 | 42 |
|  |  | 45 | M | 73.82/5.92 | 44.90/5.93 | - | 47 | 37 |
|  |  |  |  |  |  |  |  |  |
|  |  | 46 | M | 73.82/5.92 | 44.44/6.37 | - | 60 | 72 |
|  |  |  |  |  |  | phospho (ST) | 66 | 93 |
|  |  |  |  |  |  |  | 68 | 90 |
|  |  | 14 | Y | 73.82/5.92 | 59.19/5.82 | - | 69 | 45 |
|  |  |  |  |  |  | phospho (ST) | 74 | 64 |
