# Supplementary material for: Analysis of the Secretomes of Paracoccidioides Mycelia and Yeast Cells
Source: PLoS One. 2012 Dec 18;7(12):e52470. doi: 10.1371/journal.pone.0052470 (PMC3525554; doi:10.1371/journal.pone.0052470)
Supplement: Table S4 — Spectral identification of post-translational modification in identified protein/isoforms. 1 PTM – Post translational modifications: acetyl (k) – lysine acetylation; phospo (S-T-Y) – serine, threonine and tyrosine phosphorylation. 2 Peptide localization in protein sequence. 3 Theoretical peptide molecular mass without any PTM. 4 Obtained experimental mass by spectral analysis. (DOC) [file pone.0052470.s009.doc]

| Protein description | Spot number | PTM^1^ | Peptide sequence | Peptide localization^2^ | Theoretical mass^3^ | Measured mass^4^ |
| --- | --- | --- | --- | --- | --- | --- |
| hsp70-like protein | 76 | 3 Acetyl (K) | AGKPVISVEFKGEEK | 96 – 110 | 1617.86 | 1743.92 |
|  |  | Acetyl (K) | QFTPEEISSMVLTK | 111 – 124 | 1609.88 | 1651.82 |
|  |  | Acetyl (K) | QFTPEEISSMVLTKMR | 111 – 126 | 1897.27 | 1938.95 |
|  |  |  |  |  |  |  |
|  | 86 | 3 Acetyl (K) | AGKPVISVEFKGEEK | 96 – 110 | 1617.86 | 1743.96 |
|  |  | Acetyl (K) | QFTPEEISSMVLTK | 111 – 124 | 1609.88 | 1651.84 |
|  |  | Acetyl (K) | QFTPEEISSMVLTKMR | 111 – 126 | 1897.27 | 1938.99 |
|  |  | 2 Acetyl (K) | TKDNNLLGK | 449 – 457 | 1002.12 | 1086.54 |
|  |  |  |  |  |  |  |
|  | 57 | Acetyl (K) | AGKPVISVEFKGEEK | 96 – 110 | 1617.86 | 1659.89 |
|  |  | 3 Acetyl (K) | AGKPVISVEFKGEEK | 96 – 110 | 1617.86 | 1743.87 |
|  |  | Acetyl (K) | QFTPEEISSMVLTK | 111 – 124 | 1609.88 | 1651.77 |
|  |  | Acetyl (K) | QFTPEEISSMVLTKMR | 111 – 126 | 1897.27 | 1938.89 |
|  |  | Phospho (ST) | LIGDAAKNQVAMNPSNTVFDAK | 48 – 69 | 2304.6 | 2383.94 |
|  |  | Phospho (ST) | STMDPVERVLR | 310 – 320 | 1302.53 | 1382.74 |
| aconitase | 3 | Phospho (ST) | SYAHSLR | 268 – 274 | 832.92 | 913.48 |
|  |  | 2 Phospho (ST) | DRSSISVAVSPTSDR | 497 – 511 | 1576.7 | 1736.86 |
| mannitol-1-phosphate 5-dehydrogenase | 99 | Phospho (Y) | AIHWVDNLEPYIER | 199 – 212 | 1754.97 | 1834.82 |
| methylmalonate-semialdehyde dehydrogenase | 142 | Acetyl (K) | CMALSTLVMVGETKEWLPEIAER | 367 – 389 | 2607.14 | 2705.17 |
| nucleoside diphosphate kinase | 130 | Acetyl (K) | SASEQTFIAIKPDGVQR | 2 – 18 | 1847.07 | 1889.04 |
| peptidyl-prolyl cis-trans isomerase B | 159 | 2 Acetyl (K) | IQNVEKGLGDKPK | 173 – 185 | 1425.63 | 1509.90 |
|  |  | Phospho (Y) | DFMIQGGDFTNGDGTGGRSIYGEK | 90 – 113 | 2522.7 | 2602.30 |
| peptidyl-prolyl cis-trans isomerase D | 58 | Acetyl (K) | AETTGRPRVFFDIEVGNKPEGR | 2 – 23 | 2475.77 | 2517.32 |
|  |  | 2 Acetyl (K) | SGKLLSYK | 53 – 60 | 895.06 | 979.47 |
|  |  | 2 Phospho (ST) | LAPGDAAVVSETARVK | 339 – 354 | 1583.81 | 1743.75 |
| peptidyl-prolyl cis-trans isomerase H | 160 | 2 Phospho (ST) | MELFADVTPKTAENFR | 30 – 45 | 1869.15 | 2029.03 |
|  |  |  |  |  |  |  |
|  | 124 | Acetyl (K) | GKPQGYKGSK | 57 –66 | 1049.18 | 1091.57 |
| 2-methylcitrate synthase | 40 | Phospho (ST) | RGEDVIGEVTVASTIGGMR | 56-74 | 1946.99 | 2027.14 |
|  |  |  |  |  |  |  |
|  |  | Phospho (ST) | WILKMQSTIGNK | 315-326 | 1434.89 | 1514.89 |
|  |  |  |  |  |  |  |
|  | 50 | Phospho (ST) | MALASRATR | 1-9 | 976.53 | 1056.59 |
|  |  | Phospho (ST) | NSEIAPGVLAEHGKTK | 386-401 | 1650.88 | 1730.93 |
|  |  | 2 Phospho (ST) | NSEIAPGVLAEHGKTK | 386-401 | 1650.88 | 1810.91 |
|  |  | Phospho (ST) | TKNPHPNVDAASGVLFYHYGFR | 400-421 | 2490.36 | 2570.43 |
|  |  | Acet (K) | RPEIQANPVFQLVKK | 371-385 | 1767.11 | 1850.97 |
|  |  | Acet (K) | NSEIAPGVLAEHGKTK | 386-401 | 1650.88 | 1692.94 |
|  |  |  |  |  |  |  |
|  | 69 | Phospho (ST) | MALASRATR | 1-9 | 976.53 | 1056.57 |
|  |  | Phospho (ST) | NSEIAPGVLAEHGKTK | 386-401 | 1650.88 | 1730.90 |
|  |  | 2 Phospho (ST) | NSEIAPGVLAEHGKTK | 386-401 | 1650.88 | 1810.90 |
|  |  | Phospho (ST) | TKNPHPNVDAASGVLFYHYGFR |  | 2490.23 | 2570.41 |
|  |  | Acetyl (K) | FYSTAESDLK | 27-36 | 1160.54 | 1202.59 |
|  |  | Acetyl (K) | HILDLFK | 151-157 | 885.56 | 927.53 |
|  |  | Acetyl (K) | NSEIAPGVLAEHGK | 386-399 | 1421.79 | 1463.68 |
|  |  | Acetyl (K) | NSEIAPGVLAEHGKTK | 386-401 | 1650.88 | 1692.92 |
|  |  |  |  |  |  |  |
|  | 68 | 2 Phospho (ST) | NSEIAPGVLAEHGKTK | 386-401 | 1650.88 | 1810.96 |
|  |  | Acetyl (K) | FYSTAESDLK | 27-36 | 1160.54 | 1202.63 |
|  |  |  |  |  |  |  |
|  | 53 | Phospho (ST) | RGEDVIGEVTVASTIGGMR | 56-74 | 1946.99 | 2027.07 |
|  |  | Phospho (ST) | NSEIAPGVLAEHGKTK | 386-401 | 1650.88 | 1730.82 |
|  |  | Phospho (ST) | SVNLQGLLDLSRK | 457-469 | 1442.82 | 1522.82 |
|  |  | Acetyl (K) | FYSTAESDLKTVFEK | 27-41 | 1764.86 | 1806.85 |
|  |  |  |  |  |  |  |
|  | 70 | Phospho (ST) | NSEIAPGVLAEHGKTK | 386-401 | 1650.88 | 1730.93 |
|  |  | Phospho (ST) | NSEIAPGVLAEHGKTK | 386-401 | 1650.88 | 1810.92 |
|  |  | Acetyl (K) | FYSTAESDLK | 27-36 | 1160.54 | 1202.61 |
|  |  |  |  |  |  |  |
|  | 52 | Phospho (ST) | RGEDVIGEVTVASTIGGMR | 56-74 | 1946.99 | 2027.08 |
|  |  | 3 Phospho (ST) | DMHPMTQLSVAVAALNTESK | 162-181 | 2149.55 | 2399.03 |
|  |  | 2 Phospho (ST) | NYLWSTLK | 334-341 | 1024.84 | 1184.56 |
|  |  | Phospho (ST) | NSEIAPGVLAEHGKTK | 386-401 | 1650.88 | 1730.84 |
|  |  | Phospho (ST) | TKNPHPNVDAASGVLFYHYGFR | 400-421 | 2490.23 | 2570.28 |
|  |  | Phospho (ST) | SVNLQGLLDLSRK | 457-469 | 1442.80 | 1522.73 |
|  |  | Acetyl (K) | ASQSAAWSFASSRFYSTAESDLK | 14-36 | 2497.16 | 2539.29 |
|  |  | Acetyl (K) | FYSTAESDLKTVFEK | 27-41 | 1764.86 | 1806.87 |
|  |  |  |  |  |  |  |
|  | 39 | Phospho (ST) | RGEDVIGEVTVASTIGGMR | 56-74 | 1946.99 | 2027.08 |
|  |  | Phospho (ST) | WILKMQSTIGNK | 315-326 | 1434.77 | 1514.89 |
|  |  | 2 Phospho (ST) | NYLWSTLK | 334-341 | 1024.84 | 1184.56 |
|  |  | 2 Acetyl (K) | SHLPKHILDLFK | 146-157 | 1447.84 | 1531.83 |
| 6-phosphoglunconolactonase | 157 | 2 Phospho (ST) | FSTWEIFFADER | 70-81 | 1547.70 | 1707.76 |
|  |  | 2 Phospho (ST) | ITLTLPVVTHGLR | 195-207 | 1419.86 | 1579.70 |
|  |  | 2 Acetyl (K) | IAFVAMGAGKK |  | 1108.54 | 1192.65 |
|  |  |  |  |  |  |  |
|  | 102 | 3 Phospho (ST) | MVGGMPNLYTFPDTNTLAK | 1-19 | 2101.98 | 2342.03 |
|  |  | Phospho (ST) | ITLTLPVVTHGLRIAFVAMGAGK | 195-217 | 2381.36 | 2461.28 |
|  |  | Acetyl (K) | IAFVAMGAGK | 208-217 | 964.54 | 1006.52 |
| adenosine kinase | 73 | Phospho (ST) | AGLHTEYRVDETQPTGR | 104-120 | 1929.95 | 2009.92 |
|  |  | Phospho (ST) | QPHIWSLVEKAK | 149-160 | 1435.80 | 1515.75 |
|  |  | Acetyl (K) | SLCTHLAASNEYK | 131-143 | 1493.71 | 1535.77 |
|  |  | 2 Acetyl (K) | QPHIWSLVEKAK | 149-160 | 1435.80 | 1519.78 |
|  |  | Acetyl (K) | RTSVHAIAK | 267-275 | 982.57 | 1024.58 |
| thioredoxin-like protein | 100 | Phospho (ST) | NPHYRDSLQVIFR | 53-65 | 1644.89 | 1724.84 |
|  |  | Phospho (ST) | NETYARLAK | 118-126 | 1065.56 | 1145.61 |
| dihydrolipoyl dehydrogenase | 48 | Acetyl (K) | EAAMATYSKAIHF | 509-521 | 1455.68 | 1497.77 |
| dipeptidil peptidase | 18 | Phospho (ST) | LVASAESMIPKLPWSK | 413-428 | 1773.02 | 1852.97 |
|  |  | Phospho (ST) | DYRDPAFEVQVGIHELLGHGTGK | 502-524 | 2538.39 | 2618.48 |
|  |  | Phospho (ST) | YSIMRTFLDAGGDFVK | 645-660 | 1819.90 | 1900.00 |
|  |  | Acetyl (K) | TDAGNFELLIASANK | 271-285 | 1563.87 | 1605.85 |
|  |  | Acetyl (K) | AGVMALEFWDPKSSK | 621-635 | 1665.83 | 1707.85 |
| disulfide isomerase Pdi 1 | 154 | 2 Acetyl (K) | KSSSITSYMVK | 121-131 | 1246.63 | 1330.68 |
| DNA damage checkpoint protein rad24 | 64 | Acetyl (K) | GYEDAVYLAK | 2-11 | 1128.55 | 1170.55 |
| enolase | 47 | Phospho (ST) | LAFQEFMIVPTAAPSFSEALR | 164-184 | 2341.28 | 2421.34 |
|  |  | Phospho (ST) | IALDIASSEFYK | 243-254 | 1356.76 | 1436.73 |
| fructose-biphosphate aldolase | 91 | Acetyl (K) | SIAPSYGIPVVLHTDHCAKK | 96-115 | 2193.14 | 2235.17 |
|  |  | Acetyl (K) | LHPELLSKHQAYVK | 237-250 | 1662.93 | 1704.99 |
|  |  |  |  |  |  |  |
|  | 63 | Acetyl (K) | EGEKTMSAR | 338-346 | 1024.46 | 1066.49 |
|  |  |  |  |  |  |  |
|  | 89 | Acetyl (K) | SIAPSYGIPVVLHTDHCAK | 96-114 | 2065.13 | 2107.17 |
|  |  | Acetyl (K) | SIAPSYGIPVVLHTDHCAKK | 96-115 | 2193.14 | 2235.18 |
| fumarylacetoacetase | 61 | 2 Phospho (ST) | MTASSWLRIPK | 1-11 | 1289.70 | 1449.77 |
|  |  | Phospho (ST) | SNEALRSSALIPR | 106-118 | 1413.78 | 1493.76 |
|  |  | Phospho (ST) | NFATTITPWVVLMDALEPFR | 265-284 | 2321.19 | 2401.23 |
| glutamate carboxypeptidase | 44 | 3 Phospho (ST) | MPPASLPISRIAASLPPPTR | 1-20 | 2072.16 | 2312.16 |
|  |  | Phospho (ST) | NVLLLPMGSSTDMAHSTNEK | 522-541 | 2145.03 | 2225.10 |
| glyceraldehyde-3-phosphate dehydrogenase | 93 | Acetyl (K) | AVGKVIPALNGK | 216-227 | 1166.72 | 1208.62 |
|  |  | 2 Acetyl (K) | AAVKAASEGELK | 260-271 | 1173.64 | 1257.71 |
| heat shock protein 60 | 137 | 2 Phospho (ST) | GIQSAVEAVVEYLQTNK | 156-172 | 2008.98 | 2008.98 |
|  |  | Phospho (ST) | TIDDELEVTEGMRFDR | 219-234 | 2021.90 | 2021.90 |
|  |  | 2 Phospho (ST) | GYVSPYFITDTKAQK | 235-249 | 1717.87 | 1877.85 |
|  |  | 3 Phospho (ST) | GYVSPYFITDTKAQK | 235-249 | 1717.87 | 1957.89 |
|  |  | Phospho (ST) | TIVENSGLEGSVIVGK | 492-507 | 1601.87 | 1681.80 |
|  |  | Acetyl (K) | AIFSETVKNVAAGCNPMDLR | 135-154 | 2193.07 | 2235.16 |
|  |  | 2 Acetyl (K) | VEFEKPLILLSEK | 250-262 | 1544.88 | 1628.84 |
|  |  | Acetyl (K) | GQLQVAAVK | 306-314 | 913.54 | 955.47 |
|  |  | Acetyl (K) | LAKLSGGVAVIK | 407-418 | 1155.74 | 1197.66 |
|  |  | Acetyl (K) | GEYVDMIGAGIVDPLKVVR | 525-543 | 2047.08 | 2088.98 |
|  |  |  |  |  |  |  |
|  | 17 |  | TIDDELEVTEGMRFDR | 219-234 | 1941.92 | 2021.96 |
|  |  | 2 Phospho (ST) | GYVSPYFITDTKAQK | 235-249 | 1717.87 | 1877.90 |
|  |  |  |  |  |  |  |
|  | 29 | Phospho (ST) | MQRAFTSSR | 1-9 | 1099.53 | 1179.60 |
|  |  | 2 Phospho (ST) | GYVSPYFITDTKAQK | 235-249 | 1717.87 | 1877.86 |
|  |  | Acetyl (K) | GIQSAVEAVVEYLQTNKR | 156-173 | 2005.06 | 2047.07 |
|  |  |  |  |  |  |  |
|  | 42 | 2 Phospho (ST) | FDRGYVSPYFITDTK | 234-246 | 1808.92 | 1968.98 |
|  |  | 2 Phospho (ST) | GYVSPYFITDTKAQK | 235-249 | 1717.81 | 1877.92 |
|  |  | Phospho (ST) | AFTSSRASVLSSASSTR | 4-20 | 1714.87 | 1794.83 |
|  |  | 2 Acetyl (K) | VEFEKPLILLSEK | 250-262 | 1544.91 | 1628.88 |
| heat shock protein SSC1 | 7 | 2 Phospho (ST) | TTPSVVAFTKDGER | 82-95 | 1507.78 | 1667.79 |
|  |  | 2 Phospho (ST) | SQTFSTAADFQTAVEIK | 465-481 | 1843.99 | 2003.91 |
|  |  | 2 Phospho (ST) | AAIEAANRADSVLNDTEK | 573-590 | 1887.95 | 2047.95 |
|  |  | Phospho (ST) | IASLREVVAK | 611-620 | 1085.66 | 1165.73 |
|  |  |  |  |  |  |  |
|  | 46 | 3 Phospho (ST) | GQVIGIDLGTTNSAVAVMEGK | 49-69 | 2076.17 | 2316.24 |
|  |  | 2 Phospho (ST) | TTPSVVAFTKDGER | 82-95 | 1507.77 | 1667.85 |
|  |  | 2 Phospho (ST) | NGVVTVPAYFNDSQRQATK | 184-102 | 2095.05 | 2256.18 |
|  |  | Phospho (ST) | HINSKMTR | 337-344 | 986.57 | 1066.52 |
|  |  | Phospho (ST) | SQLEALVDPLISRTIEPVR | 345-363 | 2136.20 | 2216.24 |
|  |  | 2 Phospho (ST) | VGESVKSIFGR | 391-401 | 1178.65 | 1338.82 |
|  |  | Phospho (ST) | SQSGEGSITADELK | 621-634 | 1421.67 | 1500.75 |
|  |  | Acetyl (K) | GQVIGIDLGTTNSAVAVMEGK | 49-69 | 2060.06 | 2101.99 |
|  |  | Acetyl (K) | QAVVNPENTLFATK | 104-117 | 1531.90 | 1572.86 |
|  |  | Acetyl (K) | YSPSQIGGFVLQKMK | 157-171 | 1698.88 | 1782.96 |
|  |  | Acetyl (K) | EKIASLR | 609-615 | 816.49 | 858.24 |
|  |  | Acetyl (K) | SQSGEGSITADELK | 621-634 | 1421.67 | 1462.83 |
|  |  | Acetyl (K) | SEEGQQQQSQQSNEGQQGGEGEK | 656-678 | 2477.04 | 2519.45 |
|  |  |  |  |  |  |  |
|  | 14 | 2 Phospho (ST) | TTPSVVAFTKDGER | 82-95 | 1507.77 | 1667.83 |
|  |  | 2 Phospho (ST) | YSPSQIGGFVLQK | 157-169 | 1423.78 | 1583.79 |
|  |  | 2 Phospho (ST) | AAIEAANRADSVLNDTEK | 573-590 | 1887.94 | 2047.99 |
|  |  |  |  |  |  |  |
|  | 43 | Phosphor(S/T) | SSSRSSAYK | 20-28 | 972.47 | 1052.44 |
|  |  | 2 Phospho (ST) | TTPSVVAFTKDGER | 82-95 | 1507.76 | 1667.79 |
|  |  | 2 Phospho (ST) | SQTFSTAADFQTAVEIK | 465-481 | 1844.01 | 2003.92 |
|  |  | 2 Phospho (ST) | AAIEAANRADSVLNDTEK | 573-590 | 1887.94 | 2047.96 |
|  |  | Phospho (ST) | IASLREVVAK | 611-620 | 1085.66 | 1165.73 |
|  |  |  |  |  |  |  |
|  | 59 | Phosphor(S/T) | SSSRSSAYK | 20-28 | 972.47 | 1052.44 |
|  |  | 2 Phospho (ST) | TTPSVVAFTKDGER | 82-95 | 1507.77 | 1667.78 |
|  |  | 2 Phospho (ST) | YSPSQIGGFVLQK | 157-169 | 1423.75 | 1583.77 |
|  |  | Phospho (ST) | KESGIDLSNDR | 288-298 | 1233.60 | 1313.68 |
|  |  | Phospho (ST) | DIQEVILVGGMTRMPK | 375-390 | 1786.95 | 1866.92 |
|  |  | 2 Phospho (ST) | AAIEAANRADSVLNDTEK | 573-590 | 1887.94 | 2047.93 |
