# Supplementary material for: Analysis of the Secretomes of Paracoccidioides Mycelia and Yeast Cells
Source: PLoS One. 2012 Dec 18;7(12):e52470. doi: 10.1371/journal.pone.0052470 (PMC3525554; doi:10.1371/journal.pone.0052470)
Supplement: Table S5 — Comparative analysis of secreted proteins/isoforms by Paracoccidioides Pb 01 yeast cells with others pathogenic fungal secretomes. 1 Spots numbers indicated in Figure 2. 2 NCBI database general information number of Paracoccidioides Pb01 (http://www.ncbi.nlm.nih.gov/). 3 Accession number of orthologues present in the Paracoccidioides Pb18 secretome (Vallejo et al, 2012). 4 Accession number of orthologues present in the Histoplasma capsulatum secretome (Albuquerque et al, 2008; Holbrook et al, 2011). 5 Accession number of orthologues present in the Cryptococcus neoformans extracelular vesicles (Rodrigues et al, 2008). 6 Accession number of orthologues present in the Aspergillus fumigatus secretome (Wartenberg et al, 2011). (DOC) [file pone.0052470.s010.doc]

| **Spot number^1^** | **General Information Number (NCBI)^2^** | **Identified Protein in isolate *Pb*01** | **Orthologues in isolate *Pb*18 ^3^** | **Orthologues in *Histoplasma capsulatum* ^4^** | **Orthologues in *Cryptococcus neoformans* ^5^** | **Orthologues in *Aspergillus fumigatus* ^6^** |
| --- | --- | --- | --- | --- | --- | --- |
|
| 126 | gi|295660305 | cytochrome-c oxidase chain VI |  | HCAG_05938.1 |  |  |
| 96 | gi|225678203 | NmrA-like family protein |  |  |  |  |
| 114 | gi|295659831 | Thioredoxin-like protein |  |  |  |  |
| 77 | gi|295663891 | 2,5-diketo-D-gluconic acid reductase A |  |  |  |  |
| 50 | gi|295666179 | 2-methylcitrate synthase | PADG_04710 | HCAG_05090.1 |  |  |
| 51 | gi|295666179 | 2-methylcitrate synthase | PADG_04710 | HCAG_05090.1 |  |  |
| 52 | gi|295666179 | 2-methylcitrate synthase | PADG_04710 | HCAG_05090.1 |  |  |
| 39 | gi|295666179 | 2-methylcitrate synthase | PADG_04710 | HCAG_05090.1 |  |  |
| 70 | gi|295666179 | 2-methylcitrate synthase | PADG_04710 | HCAG_05090.1 |  |  |
| 69 | gi|295666179 | 2-methylcitrate synthase | PADG_04710 | HCAG_05090.1 |  |  |
| 68 | gi|295666179 | 2-methylcitrate synthase | PADG_04710 | HCAG_05090.1 |  |  |
| 53 | gi|295666179 | 2-methylcitrate synthase | PADG_04710 | HCAG_05090.1 |  |  |
| 101 | gi|295662074 | 3-hydroxybutyryl-CoA dehydrogenase |  | HCAG_07725.1 |  |  |
| 102 | gi|295663567 | 6-phosphogluconolactonase |  |  |  | |
| 37 | gi|295659859 | acetyl-CoA acetyltransferase | PADG_02751 |  |  |  |
| 19 | gi|225684009 | aconitase | PADG_04898 |  |  |  |
| 20 | gi|295673184 | actin-interacting protein |  |  |  |  |
| 8 | gi|295673184 | actin-interacting protein |  |  |  |  |
| 85 | gi|295674697 | adenosine kinase |  |  |  |  |
| 9 | gi|295667902 | aminomethyltransferase |  |  |  |  |
| 95 | gi|295667790 | beta-glucosidase |  | HCAG_01828.1 |  |  |
| 1 | gi|295664474 | cell division cycle protein |  | HCAG_02452.1 |  |  |
| 15 | gi|225680243 | Cobalamin-independent methionine synthase | PABG_07587 |  |  |  |
| 129 | gi|295658863 | Cofilin/tropomyosin-type actin-binding family protein |  |  |  |  |
| 74 | gi|295667926 | conserved protein |  |  |  |  |
| 120 | gi|295657286 | conserved protein |  |  |  |  |
| 128 | gi|295670838 | Nuclear transport factor 2 |  | HCAG_00131.1 |  |  |
| 127 | gi|295660305 | cytochrome-c oxidase chain VI |  |  |  |  |
| 34 | gi|295668473 | dihydrolipoyl dehydrogenase |  | EEH03184.1 |  | AFUA 2G02100 |
| 49 | gi|295668473 | dihydrolipoyl dehydrogenase |  | EEH03184.1 |  | AFUA 2G02100 |
| 48 | gi|295668473 | dihydrolipoyl dehydrogenase |  | EEH03184.1 |  | AFUA 2G02100 |
| 6 | gi|295666432 | dipeptidyl-peptidase | PADG_00053 |  |  |  |
| 18 | gi|295660102 | dipeptidyl-peptidase | PADG_05160 |  |  |  |
| 2 | gi|295660102 | dipeptidyl-peptidase | PADG_05160 |  |  |  |
| 21 | gi|295673162 | disulfide isomerase Pdi1 | PAAG_00986 | HCBG_00825.2 |  |  |
| 83 | gi|295672736 | DNA damage checkpoint protein rad24 | PAAG_00773 |  |  |  |
| 94 | gi|295672736 | DNA damage checkpoint protein rad24 | PAAG_00773 |  |  |  |
| 71 | gi|295661300 | DNA damage checkpoint protein rad24 | PADG _04440 | HCBG_00457.2 |  |  |
| 64 | gi|295661300 | DNA damage checkpoint protein rad24 | PADG_04440 | HCBG_00457.2 |  |  |
| 28 | gi|295672732 | enolase | PADG_04059 | HCBG_00056.2 |  | AFUA_6G06770 |
| 27 | gi|295672732 | enolase | PADG_04059 | HCBG_00056.2 |  | AFUA_6G06770 |
| 47 | gi|295672732 | enolase | PADG_04059 | HCBG_00056.2 |  | AFUA_6G06770 |
| 107 | gi|295662032 | enoyl-CoA hydratase | PADG_01209 | HCBG_00056.2 |  |  |
| 119 | gi|295674311 | eukaryotic translation initiation factor 5A |  | HCAG_06021.1 |  |  |
| 33 | gi|295668479 | formamidase | PADG_06490 | HCAG_08831.1 |  |  |
| 36 | gi|295668479 | formamidase | PADG_06490 | HCAG_08831.1 |  |  |
| 35 | gi|295668479 | formamidase | PADG_06490 | HCAG_08831.1 |  |  |
| 32 | gi|295668479 | formamidase | PADG_06490 | HCAG_08831.1 |  |  |
| 63 | gi|295671120 | fructose-bisphosphate aldolase | PADG_00668 | HCAG_00010.1 |  |  |
| 81 | gi|295671120 | fructose-bisphosphate aldolase | PADG_00668 | HCAG_00010.1 |  |  |
| 91 | gi|295671120 | fructose-bisphosphate aldolase | PADG_00668 | HCAG_00010.1 |  |  |
| 89 | gi|295671120 | fructose-bisphosphate aldolase | PADG_00668 | HCAG_00010.1 |  |  |
| 90 | gi|295671120 | fructose-bisphosphate aldolase | PADG_00668 | HCAG_00010.1 |  |  |
| 79 | gi|295658698 | fumarylacetoacetase | PABG_07391 |  |  |  |
| 62 | gi|295658698 | fumarylacetoacetase | PABG_07391 |  |  |  |
| 61 | gi|295658698 | fumarylacetoacetase | PABG_07391 |  |  |  |
| 60 | gi|295658698 | fumarylacetoacetase | PABG_07391 |  |  |  |
| 111 | gi|295667597 | G4 quadruplex nucleic acid binding protein |  |  |  |  |
| 118 | gi|295660961 | gamma-glutamyltranspeptidase |  |  |  |  |
| 44 | gi|295657201 | glutamate carboxypeptidase |  | HCAG_03543.1 |  |  |
| 38 | gi|295664022 | glutathione reductase |  |  |  |  |
| 84 | gi|295664022 | glutathione reductase |  |  |  |  |
| 67 | gi|295667577 | glutathione S-transferase Gst3 |  |  |  |  |
| 82 | gi|295658119 | glyceraldehyde-3-phosphate dehydrogenase | PADG_02411 | HCAG_04910.1 | CNAG_06699.1 |  |
| 93 | gi|295658119 | glyceraldehyde-3-phosphate dehydrogenase | PADG_02411 | HCAG_04910.1 | CNAG_06699.1 |  |
| 10. | gi|295663469 | glycosyl hydrolase |  |  |  |  |
| 42 | gi|295658865 | heat shock protein 60 |  | HCBG_08832.2 |  |  |
| 29 | gi|295658865 | heat shock protein 60 |  | HCBG_08832.2 |  |  |
| 41 | gi|295658865 | heat shock protein 60 |  | HCBG_08832.2 |  |  |
| 5 | gi|295659787 | heat shock protein Hsp88 | PADG_02785 | HCAG_007831 |  |  |
| 54 | gi|295659837 | heat shock protein SSB1 | PADG_02761 |  |  |  |
| 13 | gi|295671569 | heat shock protein SSC1 | PADG_00430 | HCAG_08176.1 |  |  |
| 14 | gi|295671569 | heat shock protein SSC1 | PADG_00430 | HCAG_08176.1 |  |  |
| 59 | gi|295671569 | heat shock protein SSC1 | PADG_00430 | HCAG_08176.1 |  |  |
| 22 | gi|295659116 | hsp70-like protein | PADG_08118 | HCBG_07920.2 | CNAG_01727.1 | AFUA_1G07440 |
| 72 | gi|295659116 | hsp70-like protein | PADG_08118 | HCBG_07920.2 | CNAG_01727.1 | AFUA_1G07440 |
| 11 | gi|295659116 | hsp70-like protein | PADG_08118 | HCBG_07920.2 | CNAG_01727.1 | AFUA_1G07440 |
| 76 | gi|295659116 | hsp70-like protein | PADG_08118 | HCBG_07920.2 | CNAG_01727.1 | AFUA_1G07440 |
| 86 | gi|295659116 | hsp70-like protein | PADG_08118 | HCBG_07920.2 | CNAG_01727.1 | AFUA_1G07440 |
| 57 | gi|295659116 | hsp70-like protein | PADG_08118 | HCBG_07920.2 | CNAG_01727.1 | AFUA_1G07440 |
| 108 | gi|295665077 | Hsp90 binding co-chaperone (Sba1) |  |  |  |  |
| 115 | gi|295665077 | Hsp90 binding co-chaperone (Sba1) |  |  |  |  |
| 92 | gi|295673937 | malate dehydrogenase | PADG_07210 | HCAG_03969.1 | CNAG_03225.1 | gb|EDP47462.1 |
| 87 | gi|295662360 | mannitol-1-phosphate 5-dehydrogenase | PADG_01372 | HCBG_08109.2 |  | AFUA_2G10660 |
| 99 | gi|295662360 | mannitol-1-phosphate 5-dehydrogenase | PADG_01372 | HCBG_08109.2 |  | AFUA_2G10660 |
| 75 | gi|295662360 | mannitol-1-phosphate 5-dehydrogenase | PADG_01372 | HCBG_08109.2 |  | AFUA_2G10660 |
| 106 | gi|295668188 | nuclear movement protein nudC | PADG_03073 |  |  |  |
| 103 | gi|295665468 | nucleic acid-binding protein |  |  |  |  |
| 113 | gi|295665468 | nucleic acid-binding protein |  |  |  |  |
| 133 | gi|295666938 | nucleoside diphosphate kinase | PADG_07524 | HCAG_00544.1 | CNAG_04577.1 |  |
| 130 | gi|295666938 | nucleoside diphosphate kinase | PADG_07524 | HCAG_00544.1 | CNAG_04577.1 |  |
| 65 | gi|295658947 | O-acetylhomoserine (thiol)-lyase |  | HCAG_07004.1 |  |  |
| 122 | gi|295663907 | peptidyl-prolyl cis-trans isomerase A2 |  | HCAG_04215.1 |  |  |
| 123 | gi|295672668 | peptidyl-prolyl cis-trans isomerase B |  | HCBG_08524.2 |  |  |
| 132 | gi|295662699 | peptidyl-prolyl cis-trans isomerase cypE | PADG_07953 |  |  |  |
| 58 | gi|295668481 | peptidyl-prolyl cis-trans isomerase D | PADG_06488 | HCAG_08833.1 |  | AFUA 2G02050 |
| 125 | gi|295672447 | peptidyl-prolyl cis-trans isomerase H | PABG_03817 |  |  |  |
| 124 | gi|295672447 | peptidyl-prolyl cis-trans isomerase H | PABG_03817 |  |  |  |
| 24 | gi|225681400 | peroxisomal catalase | PADG_00324 |  |  |  |
| 66 | gi|295669690 | phosphoglycerate kinase | PADG_01896 | HCBG_03241.2 |  |  |
| 112 | gi|295672926 | proteasome component PRE4 |  | HCAG_04107.1 |  |  |
| 16 | gi|295662174 | pyruvate kinase |  | HCAG_07781.1 |  |  |
| 56 | gi|295665666 | ribonucleoprotein |  | HCBG_01992.2 |  |  |
| 97 | gi|225683737 | spermidine synthase |  | HCAG_04999.1 |  |  |
| 104 | gi|295669402 | Mn superoxide dismutase | PADG_03387 | HCAG_01543.1 |  | AFUA 4G11580 |
| 131 | gi|295666684 | Cu - Zn superoxide dismutase |  |  | CNAG_02292.1 | AFUA_5G09240 |
| 55 | gi|295666684 | Cu - Zn superoxide dismutase |  |  | CNAG_02292.1 | AFUA_5G09240 |
| 117 | gi|295659831 | thioredoxin-like protein |  |  |  |  |
| 80 | gi|295665168 | TOS1 |  |  |  |  |
| 105 | gi|295662829 | vesicular-fusion protein SEC17 |  |  |  |  |
